# Human mobility description by physical analogy of electric circuit network based on GPS data

**DOI:** 10.1038/s41598-024-63719-z

**Published:** 2024-06-11

**Authors:** Zhihua Zhong, Hideki Takayasu, Misako Takayasu

**Affiliations:** 1 School of Computing, Tokyo Institute of Technology, 4259, Nagatsutachō, Midori Ward, Yokohama, 226-8503 Japan; 2 Sony Computer Science Laboratories, Tokyo, Japan

**Keywords:** Applied mathematics, Computational science, Information technology, Applied physics

## Abstract

Human mobility in an urban area is complicated; the origins, destinations, and transportation modes of each person differ. The quantitative description of urban human mobility has recently attracted the attention of researchers, and it highly related to urban science problems. Herein, combined with physics inspiration, we introduce a revised electric circuit model (RECM) in which moving people are regarded as charged particles and analogical concepts of electromagnetism such as human conductivity and human potential enable us to capture the characteristics of urban human mobility. We introduce the unit system, ensure the uniqueness of the calculation result, and reduce the computation cost of the algorithm to 1/10,000 compared with the original ECM, making the model more universal and easier to use. We compared features including human conductivity and potential between different major cities in Japan to show our improvement of the universality and the application range of the model. Furthermore, based on inspiration of physics, we propose a route generation model (RGM) to simulate a human flow pattern that automatically determines suitable routes between a given origin and destination as a source and sink, respectively. These discoveries are expected to lead to new approaches to the solution of urban science problems.

## Introduction

Human mobility affects basic aspects of human daily life in an urban city: economic development, energy use, and evolutions of the shape and structure of cities^1–14^. It is essential to examine traces of human activities in urban areas to control the spread of infectious diseases such as COVID-19^15,16^.

Previous studies on human mobility can be roughly categorized into two types: macroscopic and microscopic. Macroscopic research mostly focuses on non-trivial macro relationships of human mobility between cities or countries through models, such as the gravity model and the radius model^17–23^. In these models, the target of the study is the flow of people moving between two separate cities A and B. The intensity of this flow is approximated using a function, which is the product of the populations in these cities divided by the power law of the distance between those cities. This function, is based on a generalized form of Newton’s law of gravitation for the attractive force between two planets. This simple model can quantitatively describe the human flow between cities; however, it neglects most of the information on individual mobility within the cities. Regarding microscopic research, it has applied machine-learning algorithms to big GPS data with the primary aim of examining the detailed moving trajectories of individuals to predict their individualized movements within a city^24–31^.

Between macroscopic and microscopic perspective, there are studies on the mesoscopic properties of human mobility, which ignore individual movements and instead focus on the flow of people within small areas in big cities^32–34^. Recently, Shida et al.^35^ proposed a novel model of human mobility, called the electric circuit model (ECM), which is inspired by the physical structure of an electric circuit, to parse time-dependent human flow patterns in urban areas. The basic idea of the ECM is to treat each moving person as a positively charged particle and describe massive human flow as electric currents on an imaginary square lattice of an electric circuit network defined over an entire city. The values of conductivity for resistors (roads) in the circuit area (urban area), which are defined from given GPS data, can clearly describe the type of infrastructure in a city; for example, conductivities along major railways are high to support huge transport system. During rush hours in the morning and evening, strong human electric potential of opposite signs appear in the city center, whereas at noon, the currents more closely resemble random thermal noises. Although the ECM was expected to be widely applicable, it has two major shortcomings: the definition of the electric circuit is not universal and depends on the given data; the computational cost associated with resistors, which depend on the GPS data, are large and not scalable for large GPS data; and the calculation result cannot be compared with different cities’ or countries’ due to that it changes with the hyperparameter of optimization algorithms.

Therefore, we revised the ECM, introducing universal definitions, such as human ampere, human ohm, and human voltage, which can be applied to any type of GPS data in a city. And we proposed a more universal definition for human resistance to eliminate limitations of ECM. The revised electric circuit model (RECM) drastically reduces the computational costs to 1/10,000, secures uniqueness in the calculated results, enabling comparisons on the GPS data of different cities even different countries when data are available. We compared the human conductivity and potential, reflecting infrastructure level and human movement tendency respectively, between different cities to show the extension of the application range of the existing ECM. Additionally, based on RECM we proposed a new model called route generation model (RGM), which generates potential routes when provided with the origin and destination in situations with an accident or disaster area and no available transport.

Our discoveries provide new approaches to capture the characteristics of human mobility pattern in urban cities. In the future, further research related to route recommendation, natural disaster simulation, and urban design, such as research on human mobility simulation for constructing new public transport systems, can be conducted.

## Results

This section describes the performance of our models, using the greater Tokyo area, which is one of the largest metropolitan cities in the world and contains one-fourth of the Japanese population (Data description shown in “Data source”). Section “Observation of human current” introduced the inspiration of human current from physics. Section “Improvement in the RECM” presents the results of the improvements accompanying the RECM. Section “Comparison between different cities” compares the human conductivity and potential between other different cities in Japan. Finally, section “Route generation model” describes the performance of the RGM and a proposed application to simulate human flow patterns in a situation where traffic is blocked in a part of the city owing to an accident or disaster.

### Observation of human current

**Figure 1:**
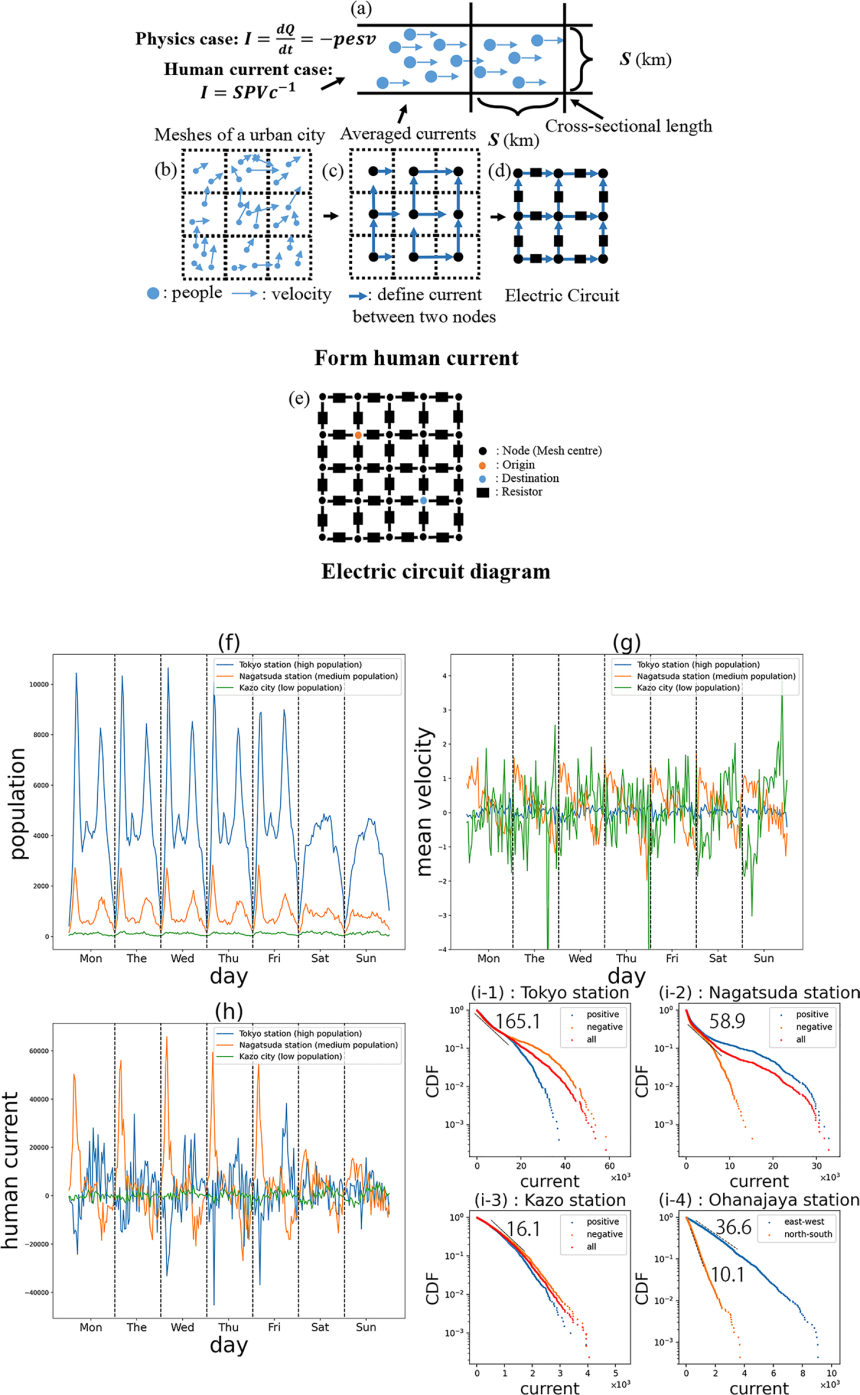
Human current. (**a**–**e**) Illustration of the working of the model. (**f**–**h**) Time series of the population, mean velocity, and human current (blue, orange, and green lines represent Tokyo Station with the high population in the city center, Nagatsuda Station with the medium population, and Kazo with the low population in the rural area from Sep 5 to Sep 9, 2022), respectively. (**i**) Human current cumulative distribution with respect to different directions in semi-log plot. (**i-1, i-2**, and** i-3**) The red, orange, and blue lines represent the distributions of the absolute, positive (direction right), and negative (direction left) values of human current, respectively. (**i-4**) The blue and orange lines represent the distribution of the absolute value of human current in the right and up directions, respectively. The guide lines show the average slopes when approximated by exponential distributions.

In electromagnetics, a current $i$ through a cross-sectional area $s$ of a metal conductor is given by $i = - \;espv$, where $p$ is the number density of the moving electrons with charge $- \;e$, and $v$ is the mean velocity component perpendicular to the cross-section. Therefore, our basic idea is to consider that moving people, carrying positive unit charges, forms human current. We defined the human current through a cross-sectional line of length $S$ as follows (see Fig. 1a):

1 $$I = SPV/c$$

where $P$ is the population density of moving people observed in the GPS data; $V$ is the mean velocity component perpendicular to the line averaged over the moving people; and $c$ is the coverage of the GPS data that was defined as the number of users in the database $N_{data} = 2,957,390$ divided by the total population of Japan $N_{total} = 124,490,000$ in 2022. Eq. (1) represents the estimated total number of people, including non-GPS users, moving through the cross-sectional line in unit time estimated from the observable GPS users. We measured the length in terms of [km] and the speed in terms of [km/h]; so the unit of the number density is [1/km^2^] and that of the human current is [1/h], which we referred to as a human ampere [HA].

For the subsequent data analysis, the city map was divided into square meshes with length $S = 0.5$ [km], as shown in Fig. 1b. Human current was defined on the links connecting the centers of neighboring meshes (Fig. 1c), and correspondingly, we introduced an electrical circuit network covering the whole city, as shown in Fig. 1d, e. Detailed calculation processes are shown in section Method; notation used in this study will be introduced in section “Notation”; and main results will be shown in the following section.

### Improvement in the RECM

By calculating the population time series over one week at typical locations with high (blue), medium (orange), and low (green) populations, Fig. 1f shows clear circadian rhythms representative of people in morning and evening rushes, except on weekends. The mean velocity time series of the people appeared in these three locations is shown in Fig. 1g. In the case of the high population mesh, which is located in the city center, the mean velocities were small because the directions of movements were scattered; in the case of the medium population mesh, which is located in a suburban residential area, the velocity profile showed a clear circadian rhythm, owing to people moving toward the city center in the morning and in the reverse direction in the evening. As defined in section “Definition of variables” Eq. (2), the human currents were calculated as the average number of products of population and mean velocity in two adjacent meshes, as shown in Fig. 1h. Strong currents and periodicity were confirmed in the medium population mesh, especially on the mornings of weekdays.

**Figure 2:**
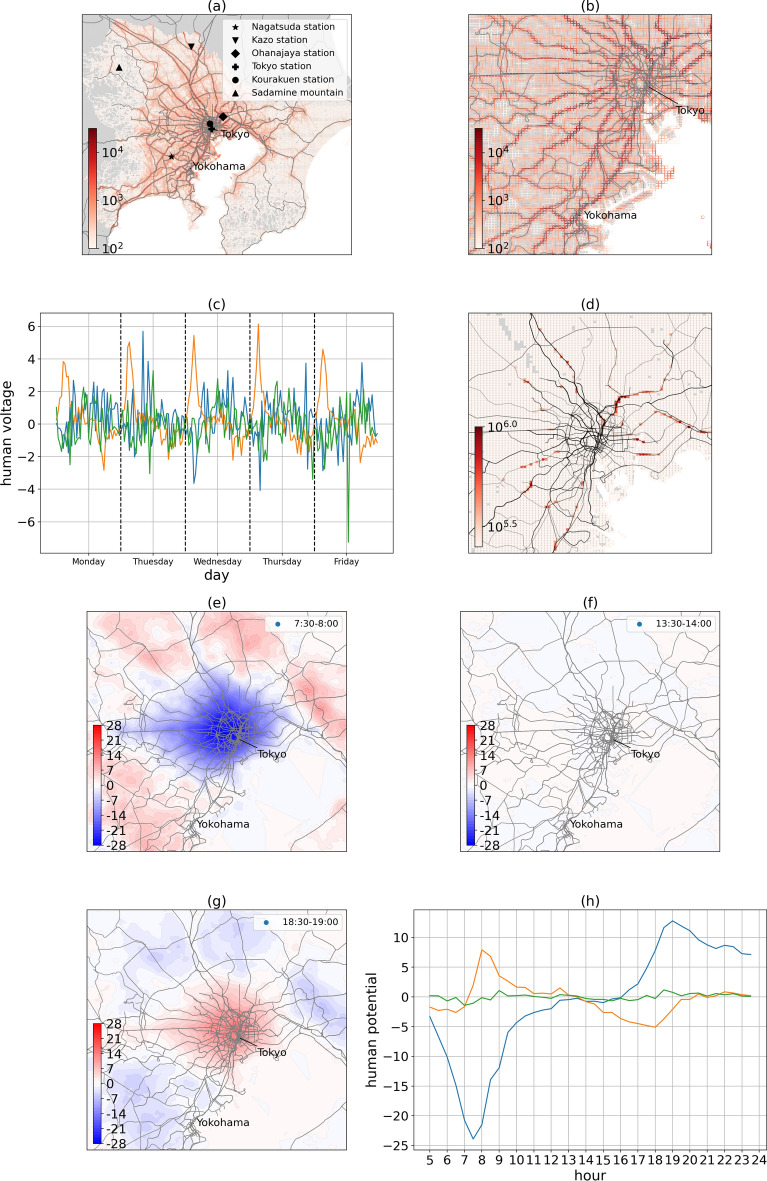
Revised electric circuit model (RECM). (**a**, **b**) Spatial distribution of the human conductivity in the greater Tokyo area. (**c**) Human voltage time series from Sep 5 to Sep 9, 2022 (the blue, orange, and green lines represent Tokyo Station with the high population in the city center, Nagatsuda Station with the medium population, and Kazo with the low population in rural area, respectively). (**d**) Spatial distribution of electrical energy dissipation which characterizes highly congested currents (time period is from 7:30 a.m. to 8:00 a.m. in the morning rush hour). (**e**–**g**) Human potential patterns at morning, noon, and evening. (**h**) Human potential time series on Sep 1, 2022.

**Figure 3:**
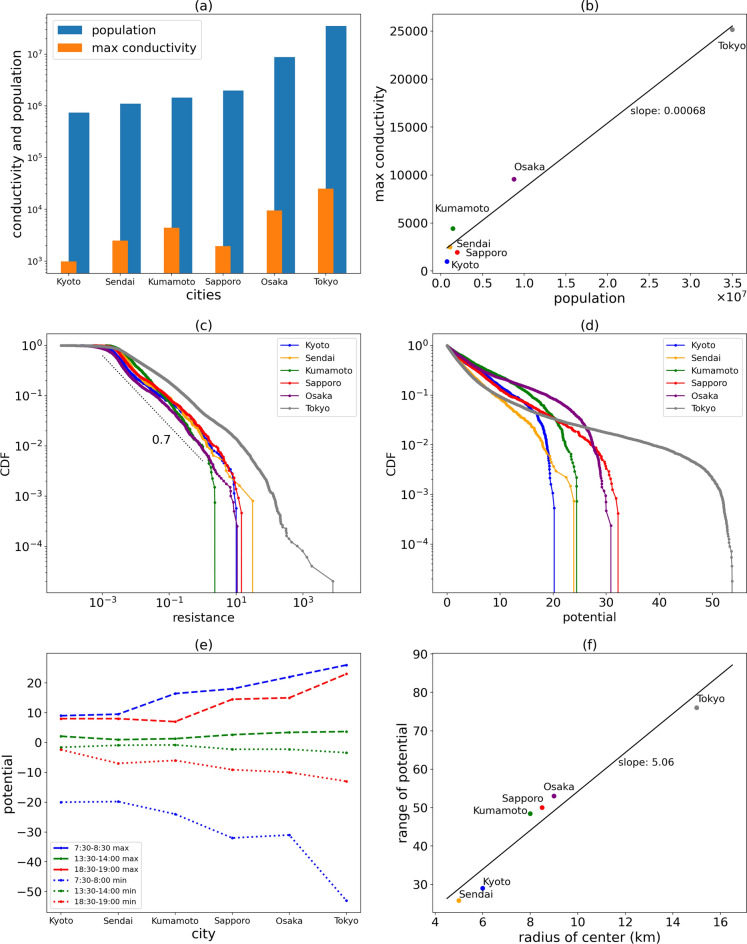
Comparison between different cities. (**a**) Bar plot of population and maximum conductivity in each city (population data is collected from: Tokyo Metropolitan Government. 2023. https://www.metro.tokyo.lg.jp/english/index.html). (**b**) Linear regression analysis on population and conductivity. (**c**, **d**) CDF of human resistance and observed potentials (in morning rush hour from 7:30 am to 8:00 am) in each city. (**e**) The maximum and minimum potential values for different periods in each city. (**f**) Linear regression analysis on the average radius of each city and the range of potential values (calculated in the morning rush hour from 7:30 am to 8:00 am).

**Figure 4:**
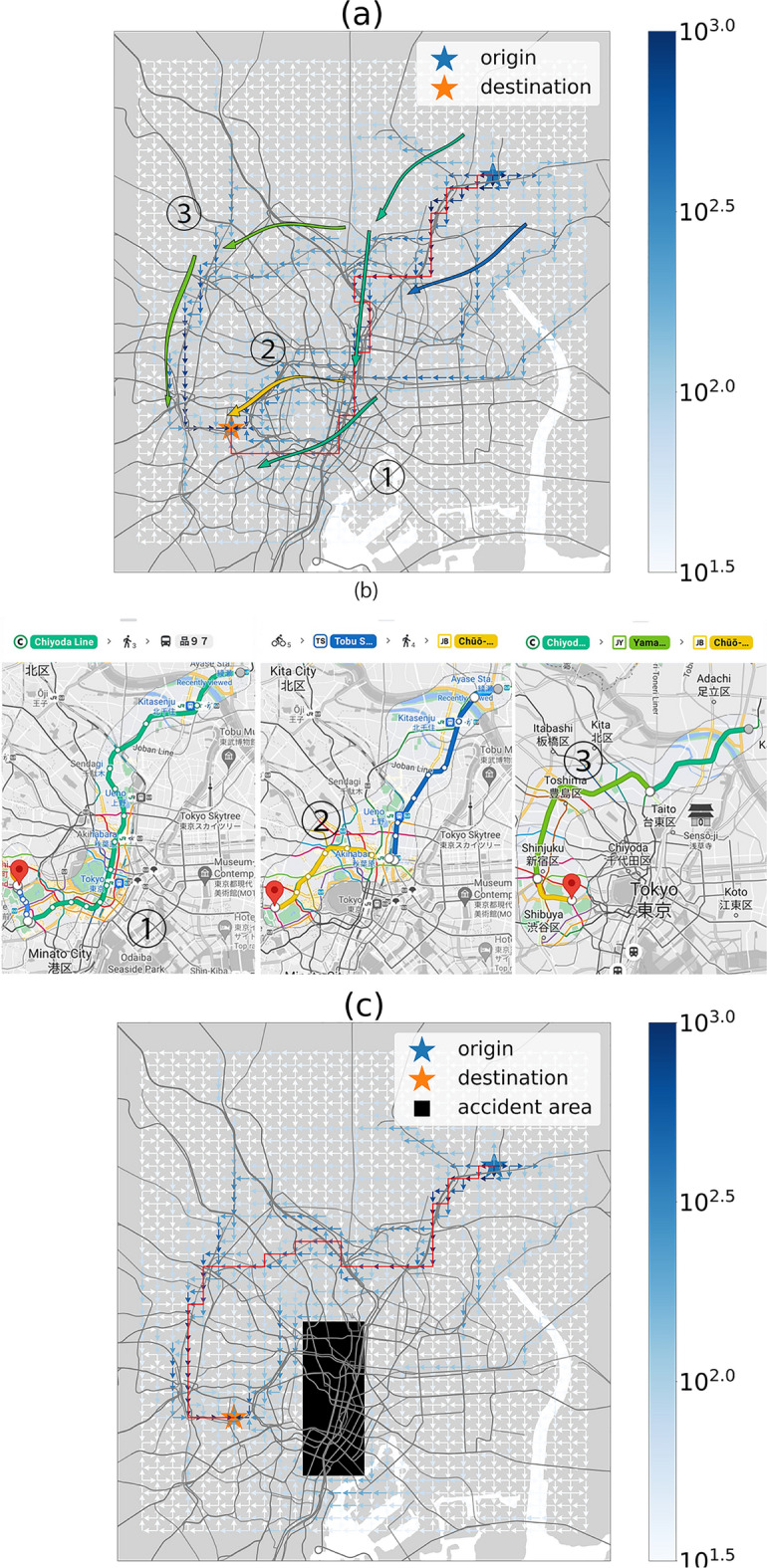
Route generation model. (**a**) Routes generated by the RGM (the origin and destination are Ayase Station and Shinanomachi Station, respectively, which are typical stations located at north-east and south-west area of Tokyo, respectively). (**b**) Routes recommended by Google Maps (Google Map. Recommendation route from Ayase station to Shinanomachi station. 2023. https://www.google.com/maps/dir/Ayase+Sta.,+3+Chome-1+Ayase,+Adachi+City,+Tokyo/Shinanomachi,+Shinjuku+City,+Tokyo/@35.7000431,139.7195152,13z/data=!4m14!4m13!1m5!1m1!1s0x60188fb9cc8beb47:0x48450c07579a074!2m2!1d139.8254234!2d35.7622297!1m5!1m1!1s0x60188c92379c0951:0x907dd02bc4813974!2m2!1d139.7198607!2d35.6817841!3e3?entry=ttu). (**c**) Routes generated by the RGM when the accident-affected area is set up (when a natural disaster occurs, and the railway or highway cannot function normally, it can be viewed as an accident-affected area, which is represented by the black rectangle in the figure; in this case, the resistance value near the accident-affected area will be set to infinity, which is a large number).

Figure 1i shows the distribution of human current for 4 different places every 0.5 h from 5 a.m. to 24 p.m. for weekdays in 2022. The positive and negative values were both plotted separately and also together for i-1, i-2, and i-3 in the semi-log scale. In each case, the plot was approximated using an exponential distribution. Fig. 1i-4 shows the case where the distribution of currents for the north–south and east–west directions followed exponential distributions with different mean values owing to the direction of the railway.

As mentioned in introduction, to solve the limitations of computational cost and not unique calculation result of resistance and potential, we proposed a new definition for human resistance, as defined in Method Eq. (4), to solve these two drawbacks simultaneously (details improvement about time complexity discussed in Method; since it was not treated as optimization problem anymore that results are sensitive to initial value and hyperparameter, uniqueness of calculation was achieved, whose details will be discussed in Supplementary Material 2). Furthermore, quantities with unclear practical meaning calculated by the black-box optimization algorithms in the previous research can be explained clearly in our study after we parsed its mathematical formula and built up its mathematical foundation.

Human conductance (Eq. (5)), $\rho_{{\left( {d,t,L,\sigma } \right)}}$, which is the inverse value of human resistance, is the slope of dashed lines in Fig. 1i. In Fig. 2a, b, the spatial distribution of human conductance is high near the railway and high-speed road, while it is low near the mountainous and rural areas. Human resistance and human conductivity reflect the ease of human flow and indicate the infrastructure level of a location, as discussed in Method.

As defined in Eq. (6), human voltage characterizes the amount of human current normalized by the average of absolute current at the same location. As known from the distributions in Fig. 1i, human voltage typically takes a value around 0, and the range of value is practically between [− 10, 10]. As shown in Fig. 2c, human voltage time series for a week showed that the maximum voltage had been approximately 4 in the mesh including Nagatsuda Station (living area) on Monday located in a suburb, implying that the current value had been approximately four times the average absolute current at that station during rush hour.

Moreover, as defined in Eq. (7), human potential showed that human behaviour resembles that of charged particles, which tend to move from a higher potential area to a lower potential area. Figure 2e–g shows the spatial pattern of human potential in the morning rush hour (7:30–8:00), noon (13:30–14:00), and evening (18:30–19:00) respectively, which also meant that we had also successfully reproduced the human potential spatial distribution after changing the definition of human resistance (Detailed discussion in Supplementary Material 2).

We can learn the tendency of how people move and distinguish where are sink and source in an urban city by observing human potential in different time period. In Fig. 2h, the human potential time series within a day at the Tokyo (blue), Nagatsuda (orange), and Kazo (green) stations showed that Tokyo Station, which has many business and commercial areas nearby, was a sink in the morning and a source in the evening. In contrast, Nagatsuda Station, which has many nearby residential areas, was a source in the morning and a sink in the evening. Since the road near Kazo Station had normal traffic flows and no residents or business areas nearby, people tended to pass through it, resulting in the human potential being close to zero all day. From this perspective, zero potential area corresponds to inactive human mobility area. It would be an interesting application of this result to detect the boundary of a city by finding out zero potential areas during rush hour.

In Fig. 2d, electrical energy dissipation, as defined in Eq. (8), shows that in the morning rush hour, high energy dissipation meshes shown in red are not in the city center but located around the suburban areas indicating that large accumulation of people occurs in these areas during morning rush hour.

Overall, human behavior in an urban city can be characterized by analogized concepts of electromagnetics, such as human current [HA], resistance [HΩ], and potential [HV]. And based on these quantities, auto classification of areas in the urban city by using unsupervised algorithm such as k-means clustering can be conducted in the future research, since time series pattern of them are quite different between resident, business, commercial, city boundary, and rural areas. Furthermore, since we made the application range of ECM more universal by securing the uniqueness of the calculation result, comparison of these quantities between different countries will also be interesting as long as other researchers have the corresponding GPS data in other countries.

### Comparison between different cities

The calculation result of the former ECM depends on the fine-tuning of the parameter of ADAM, so the result of human resistance varies and cannot be directly compared between different cities. Since the calculation of human voltage, charge, and potential is based on the resistance value, the human potential value was also parameter-dependent, and not unique. In other words, the former ECM could only be used once in one city, and the application range was limited. Thanks to the improvement of our model by changing the calculation method of the resistance value, we ensured the uniqueness of the calculation result, which enabled us to compare the characteristics in RECM between different cities in Japan.

As shown in Fig. 3a, the bar plot of population and maximum human conductivity between 5 cities, including Tokyo, Osaka, Kyoto, Sendai, and Kumamoto, shows that the infrastructure level of a city grows with the population. When more workers enter a city, the population density will increase, and better infrastructure will need to be built to support the human movement of many people. Figure 3b shows the linear relation between population and maximum conductivity (best infrastructure level) of a city; linear regression was used to determine the slope, $k = 0.0007$, which means that per 1400 people, on average, 1 unit of maximum conductivity increases.

Figure 3c shows the cumulative distribution function of human resistance for the 6 cities in log–log scale, and we found that these distributions are approximated by a power law with an exponent of about − 0.7. Figure 3d shows the distribution of absolute values of human potential for all times and places in each city in the morning rush hour from 7:30 am to 8:00 am. Tokyo, as Japan’s largest metropolitan area, has the greatest potential value; followed by Sapporo and Osaka, and then, Kumamoto, Sendai and Kyoto.

Figure 3e compares the maximum and minimum values of the potential in each city observed in 3 different time zones, morning, noon and evening. For all cities, the difference between the maximum and minimum potential values in the morning rush hour is about twice as large as that in the evening rush hour, because the time to return home in the evening has a wider range than the time to arrive at work in the morning, and major workplaces are concentrated in the city center, but residences are dispersed throughout the area, so the concentration of people flows is very different in the morning and in the evening. On the other hand, there is no clear directional flow at noon, the human flow pattern is close to random noise, and the maximum and minimum potential values are close to zero in all cities.

We now define the center of human potential for each city as the place with the minimum potential value in the morning rush hour, and then define the effective radius of the city as the average of the minimum and maximum lengths from the potential center to the boundary with zero potential. It turns out that the radius is approximately proportional to the range of the potential difference in the morning rush hour, namely, the highest voltage minus the lowest, as shown in Fig. 3f. This result is consistent with the intuition that the range of the potential difference is given by the path integral from the lowest point to the highest point, and if the slope of the local potential is roughly constant, then the effective radius of the city determines the maximum potential difference.

### Route generation model

In the RECM, temporal human potential can describe the tendency of the people in an urban city to exhibit macro movements. However, it cannot be used to generate a microscopic movement route for each individual. Herein, based on RECM, we propose a new model, RGM, that can generate possible routes for people when given specific origins and destinations. Based on the physical architecture of the electric circuit, the flow pattern of human current from the origin to the destination can be obtained by solving Kirchhoff's law^36^ under the condition that electrodes are placed at the origin and destination points.

As shown in Fig. 4a, Ayase Station and Shinanomachi Station in Tokyo were set as the origin and destination, respectively. A unit voltage was applied between these points, which were approximately 25 km apart, in a straight line. Then, the human current pattern was calculated. From the result, the most popular route (as indicated by the red line), was constructed by connecting the largest current flow direction at each mesh from the origin based on the greedy algorithm. Figure 4b shows the recommended route from Ayase Station and Shinanomachi Station provided by Google Maps. Not only the largest flux route, but also the second and third largest flux routes as green, yellow and yellow-green lines could be found; interestingly, the routes generated by RGM were consistent with the real-world paths suggested by Google Maps. Unlike traditional shortest-path algorithms, such as A* and Dijkstra^37^, the algorithms used in Google Maps that can only generate a single path from the origin to the destination to find a local solution, our model uses the global information of the system to generate all the possible paths in an electric circuit network. Therefore, ideas such as route recommendation through the development of software or smartphone applications that merge our model with traditional algorithms used in Google Maps can also be considered worthy of further research (other examples of route generation are shown in Supplementary Material 3).

Another application of our model is to predict how people will move in the event of a disaster. We assumed a situation where the trains around Tokyo Station were not operating due to a disaster such as a power outage or an earthquake. For this simulation, the conductivity of the link in the disaster area was set to 0 (black rectangle), as shown in Fig. 4c, and the model successfully provided solutions for any given origin or destination. In the case where the origin and destination were the same as those in Fig. 4a, the most popular route was indicated by the red line, which coincided with the third most popular route to avoide the disaster area. In this way, the simulation of human flow patterns before the occurrence of natural disasters or accidents for better preparation is also an interesting and important future application for the government.

## Discussion and conclusion

In this study, we showed that human behavior resembled electrically charged particle behavior and that the RECM was a powerful tool for research on human mobility. We introduced universal definitions for the existing ECM and extended its application range, as discussed in sections “Observation of human current” and “Improvement in the RECM”; compared human conductivity and potential between different cities in section “Comparison between different cities”; and proposed a new model RGM to realize the generation of possible detailed routes between a given origin and destination, as detailed in section “Route generation model”.

Regarding alternative databases, Zhang et al.^54^ showed that the population time series pattern differs between smart card data and taxi data, implying that human current patterns may vary on different datasets (e.g., GPS, smart cart, taxi). We recommend GPS data because it contains the most granular information on trip demand for individuals; however, taxi, bus, and car-navigator data are also possible alternatives since long-distance travel requires transportation tools for most individuals, and short-distance trips will not contribute much to human current regarding velocity. Information loss might exist, but similar human resistance and potential patterns should be reproduced since they mainly rely on urban structure (e.g., transportation network topology) and trip demand. Compared to different sources of location data, different groups of people should contribute more to the different mobility patterns. If location-tagged data from social media such as Twitter can be obtained, users can be classified into young, middle, and old user groups by combining the biography information. The mobility pattern of each age group shall be different because of the different trip demands. For example, children and elders tend to move between schools/parks and their homes, while middle-aged people usually commute to the city center to work. Comparing different mobility patterns for different age groups combined with socioeconomic data will also be an interesting direction for future research.

Our model has some limitations. First, the RGM does not consider time, and the best routes to travel to a particular destination may differ over time; therefore, the accuracy of the model may differ when time is considered. Second, the model does not take into account competitive relationships that if everyone uses their mobile phones to check the best route and follows the same route, traffic congestion will occur, making the recommended route inappropriate. So, competitive relationships need to be considered. Thirdly, the result of the RECM analysis is based on trip, not activity. It can describe human mobility but not accessibility, and the latter needs to integrate land use and point-of-interest (POI) data. Fourthly, as mentioned in research^61^, market share of mobile phone will also influence the statistical analyzed result.

Regarding the spatial structure of the city, there were parametric^52^ and non-parametric^53^ approaches to detect urban structure. Zhong et al.^55^ defines a centrality index by combining the visitor index and diversity index for Singapore, showing a city center and multiple subcenters. Taubenböck et al.^56^ employed a threshold-based method to detect city center in German cities in the perspective of built dimension. In our research, the human potential may be an index to show spatial structure on the morphological dimension, providing a new perspective regarding city boundaries. In Fig. 2e–g, in the morning rush hour, nodes with a potential close to zero show inactive mobility; those areas can be regarded as the boundary for Tokyo metropolitan. Note that the spatial structure of the functional dimension is highly related to land use and human daily activities. Therefore, future research can combine POI data for integral diversity information of a location with our model to build up a new centrality index for urban cities.

Fukuda et al.^51^ also showed that Tokyo is a monocentric city and concluded that if the rent is not sensitive to population growth, that population grows more in central regions; otherwise, the population grows more in suburban regions. More specifically, Fukuda et al.^51^ found that the population grew more in suburban regions until the mid-1990s, but it grew more in central regions of Tokyo after that. This opinion is also consistent with the Fujita-Ogawa model^57,58^, which considers the number of individuals residing at location $x$ and working at $y$ as proportional to the wage earned at $y$, and inversely proportional to the rental cost at $x$ and transportation cost between two places. When the wage and the transportation cost remain unchanged, the higher rental costs in the city center will make individuals tend to live in the suburban regions.

Regarding future research, the applications of our proposed models related to urban design should be wide. For example, when building a new railway or station, changes in human mobility can be assessed by reducing the resistance value of links where a new railway is planned and solving RGM to simulate changes in human mobility to quantitatively evaluate the effect of adding or removing some infrastructure at certain locations.

As shown in Fig. 4c, when a disaster occurs near Tokyo Station, and people are temporarily unable to pass through, we learned that people would use another transportation option, i.e., the Yamanote Line (Route No. 3), to bypass the area affected by the disaster. In this way, further research on the natural disaster simulator would be both interesting and valuable^38–42^. Importantly, if the government is well informed about how people move during natural disasters, it can make more effective preparations, such as building appropriate infrastructure in specific areas prone to such disasters.

As for the opposite case of evacuation, crowd gathering prediction also attracts the attention of researchers. Huang et al.^59^ computed the Z-score for the traffic volume of the links in a mobility network and detected abnormal crowds gathering activities by judging whether the score was larger than a threshold. Fan et al.^60^ clusters individual location time series into different clustering, called momentum, then employs the Markov model to predict the next location for individual trajectory. The momentum computed in their experiment has a similar effect as our human potential to express collective spatial–temporal movement trends. In our model, trip demand needs to be estimated first to predict crowd-gathering activities. For example, a big event will be held in the city center, and participants are anticipated to come from different origins. Set up their corresponding origin–destination and solve RGM multiple times, generating movement tendency (current value) and possible routes. Then, the place that may become crowded can be estimated by considering the superposition (summation) of the absolute value of the generated current for each person on the links.

Overall, combined with the inspiration of physics, we built up new models, promising to evolve into the means to solve urban problems. We hope that our contribution can be used by other researchers, such as by comparing different human flow patterns between different countries.

## Methods

In this section, we summarize the information that is necessary to implement our methods for those researchers who have access to individual GPS data. Section “Data source” introduces the data we used in this study, and any researcher who has GPS data fulfils the format of the GPS data example shown in Supplementary Material 1 can implement our models to conduct further research immediately. Section “Notation” includes the notation used in this study. Section “Definition of variables” describes the theoretical definition for quantities, analogical concept from electromagnetics, used in this study. Section “Detailed calculation procedure” illustrates the detailed procedure for practical computation, including data preprocessing (**Step 1**), calculations for the RECM (**Step 2**), and calculations for the RGM (**Step 3**). Finally, section “Time complexity of the algorithms” simply analyses the improvement of the time complexity of the algorithms, and shows that in terms of numerical calculation, it is feasible to deploy to the practical problem.

### Data source

A Japanese company, Agoop Corp., provided the database used in this study (an example of the data can be found in Supplementary 1). It contains detailed GPS data (including parameters such as user ID, time, longitude, latitude, speed, and angle) collected from over one million smartphone users in Japan from 2022/3 to 2022/11. We focused on the weekday data because the human mobility patterns differed on weekends, as previously discussed in result section.

For privacy protection reasons, the GPS data used in this research were obtained with the consent of smartphone application users. These data were anonymized in advance in accordance with the guidelines set by the general incorporated association Location Based Marketing Association (LBMA) regarding the use of location information data in Japan. Since information such as the name, address, gender, and age of a user was not included, and each user ID renewed at midnight, it was infeasible to identify individuals from just their user attributes or track their cyclical movements across days.

Overall, no privacy issues were faced when conducting this study. Approximately one billion pieces of data were available daily, with the database totalling a size of 22 TB for 2022. Regarding the computational environment, the CPU is 2 × Intel Xeon Gold 6248R (24 core); the memory is 500 GB; and the OS is Linux Ubuntu.

### Notation

As shown in Fig. 1e, each node of the electric circuit network has four links pointing in the right (east), up (north), left (west), and down (south) directions. In the system, the location of each node is represented by a set of integers (*x*, *y*), *x*, *y* ∈ *N*. To express directions, we defined a set of linear maps as $\sigma \in \left\{ {\sigma_{ + x} ,\sigma_{ + y} ,\sigma_{ - x} ,\sigma_{ - y} } \right\}$, where $\sigma_{ + x} :\left( {x,y} \right) \to \left( {x + 1,y} \right)$, $\sigma_{ + y} :\left( {x,y} \right) \to \left( {x,y + 1} \right)$, $\sigma_{ - x} :\left( {x,y} \right) \to \left( {x - 1,y} \right)$, and $\sigma_{ - y} :\left( {x,y} \right) \to \left( {x,y - 1} \right)$. Therefore, $\sigma_{ + x} \sigma_{ - x} = 1$, $\sigma_{ + y} \sigma_{ - y} = 1$, and $- \sigma : = \sigma^{ - 1} \in \left\{ {\sigma_{ - x} ,\sigma_{ - y} ,\sigma_{ + x} ,\sigma_{ + y} } \right\}$ (inverse mapping). The terms $\sigma_{ + x} ,\sigma_{ + y} , \sigma_{ - x}$, and $\sigma_{ - y}$ expressed the directions right, up, left, and down, respectively. Furthermore, $\sigma_{0} = 1,{ }\sigma_{1} = { }\sigma_{ + x} ,{ }\sigma_{2} = \sigma_{ + y} ,{ }\sigma_{3} = \sigma_{ - x}$, and $,{ }\sigma_{4} = \sigma_{ - y}$ were used for expressing rotation, which will be discussed later. For example, $I_{{\left( {d,t,L,\sigma } \right)}}$ is the human current value on day d, at time period t, in location $L$ ($L = \left( {x,y} \right)$), and in direction $\sigma$ (note that the current is defined on the link connecting node $L$ and the neighboring node $\sigma \left( L \right)$).

### Definition of variables

#### Human current

According to the architecture of the lattice electric circuit network shown in Fig. 1d, there are four link pointing to four different direction for a node. Human current $I_{{\left( {d,t,L,\sigma } \right)}}$ of the link pointing in the right direction can be defined by the following equation, which is equivalent to Eq. (1) (the basic inspiration and idea we mentioned in section “Observation of human current”), averaged over two neighboring meshes in the pointing direction:

2 $$I_{{\left( {d,t,L,\sigma_{ + x} } \right)}} = \frac{{\left( {v_{{\left( {d,t,L,\sigma_{ + x} } \right)}} *p_{{\left( {d,t,L} \right)}} + v_{{\left( {d,t,\sigma_{ + x} \left( L \right),\sigma_{ + x} } \right)}} *p_{{\left( {d,t,\sigma_{ + x} \left( L \right)} \right)}} } \right)}}{2}*\frac{1}{c}\;\;[{\text{HA}}]$$

where $p_{{\left( {d,t,L} \right)}}$ denotes the density of people with non-zero velocities, who appeared in the mesh of location $L$, at day *d*, in time period [*t*,* t* + 0.5 h]. From the data, the population density was calculated by multiplying the number of moving people (with non-zero speeds) in each mesh every 30 min with the normalization factor $1/S^{2}$, where $S = 0.5 \;\;{\text{km}}$, the length of mesh. $v_{{\left( {d,t,L,\sigma_{ + x} } \right)}}$ is the mean value of the $\sigma_{ + x}$ direction components (pointing right) of the velocities of those people in the same location and time period. Human currents in the up direction can be calculated by replacing $\sigma_{ + x}$ to $\sigma_{ + y}$; and other directions can be similarly defined. In the study, current was divided by coverage to proportionally quantify the flow of people that could not be directly observed. Following the definition of current, the following relationship holds between neighboring currents in opposite direction:

3 $$I_{{\left( {d,t,L,\sigma } \right)}} = - I_{{\left( {d,t,\sigma \left( L \right), - \sigma } \right)}}$$

#### Human resistance

Regarding to human resistance $R_{{\left( {L,\sigma } \right)}}$ [HΩ], which take time-invariant values reflecting the transportation infrastructure of each place, in the previous study^35^, resistances were determined such that the rotation of the static electric field would be close to 0, which is the condition fulfilled by a static electric circuit. However, in our study, the values of the resistances were found to be proportional to the inverse of the maximum absolute value of currents. Since the maximum was not a statistically stable quantity, we checked several candidates, as discussed in Supplementary Material 2, and concluded that the following definition of human resistance would be much easier to calculate from the data and interpret, and that approximately rotation-free electric fields, fluctuation dissipation theorem, and human potential spatial distribution could also be reproduced:

4 $$R_{{\left( {L,\sigma } \right)}} = \frac{1}{{\mathop {{\text{mean}}}\limits_{d,t} \left\{ {\left| {I_{{\left( {d,t,L,\sigma } \right)}} } \right|} \right\}}} \;\;[{\text{H}}\Omega ]$$

#### Human conductivity

Human conductivity is simply defined as the inverse of the resistance by the following formula:

5 $$\rho_{{\left( {L,\sigma } \right)}} = \frac{1}{{R_{{\left( {L,\sigma } \right)}} }} = \mathop {{\text{mean}}}\limits_{d,t} \left\{ {\left| {I_{{\left( {d,t,L,\sigma } \right)}} } \right|} \right\}$$

By combining Eqs. (2) and (5), it is easy to show that when population $p_{{\left( {d,t,L} \right)}}$ is fixed, $\rho_{{\left( {d,t,L,\sigma } \right)}}$ will only increase when $v_{{\left( {d,t,L,\sigma } \right)}}$ increases (faster transportation). On the contrary, when the velocity $v_{{\left( {d,t,L,\sigma } \right)}}$ is fixed, $\rho_{{\left( {d,t,L,\sigma } \right)}}$ will only increase when $p_{{\left( {d,t,L} \right)}}$ increases (the capability to transport people simultaneously at a given moment). Therefore, human conductivity and resistance reflects infrastructure level of a place in a city.

#### Human voltage

According to Ohm’s law, human voltage $E_{{\left( {d,t,L,\sigma } \right)}}$ [HV] on a link is simply defined by the following formula as an analogical concept:

6 $$E_{{\left( {d,t,L,\sigma } \right)}} = I_{{\left( {d,t,L,\sigma } \right)}} *R_{{\left( {L,\sigma } \right)}} = \frac{{I_{{\left( {d,t,L,\sigma } \right)}} }}{{\mathop {{\text{mean}}}\limits_{d,t} \left\{ {\left| {I_{{\left( {d,t,L,\sigma } \right)}} } \right|} \right\}}}\;\;[{\text{HV}}]$$

#### Temporal human potential

According to Helmholtz's theorem, given the spatial distribution of human voltages at a given time period, the vector field of the electric field can be decomposed into a scalar potential component without rotation and a vector potential component without divergence. In this research, the temporal human potential $\emptyset_{{\left( {d,t,L} \right)}}$ [HV] on the node of all meshes was defined by solving the Poisson equation in a discrete case as follows:

7 $$\mathop \sum \limits_{j} \Delta_{ij} \cdot \emptyset_{{\left( {d,t,L = j} \right)}} = div\left( {E_{{\left( {d,t,L = j} \right)}} } \right)$$

where $\Delta_{ij}$ is the Laplacian matrix^43–47^ of the electric circuit network, and human electric charge can be calculated by the divergence of voltage $div\left( {E_{{\left( {d,t,L = j} \right)}} } \right)$.

#### Electrical energy dissipation

According to Joule’s Law, electrical energy dissipation is defined using the following formula as an analogical concept:

8 $$W_{{\left( {d,t,L,\sigma } \right)}} = I_{{\left( {d,t,L,\sigma } \right)}}^{2} *R_{{\left( {L,\sigma } \right)}} = \frac{{I_{{\left( {d,t,L,\sigma } \right)}}^{2} }}{{\mathop {{\text{mean}}}\limits_{d,t} \left\{ {\left| {I_{{\left( {d,t,L,\sigma } \right)}} } \right|} \right\}}}$$

### Detailed calculation procedure

#### Step 1 (Data preprocessing part):

**Step 1-1.** Determine the observation day *d* (neglect weekends), divide a day into 38 time periods *t* per 30 min from 5 a.m. to 24 p.m. (for example, 5:00–5:30, 5:30–6:00, …, 23:30–24:00), and remove GPS data records with a velocity of zero or missing value (in this study, day *d* was from March 2022 to November 2022).

**Step 1-2.** Cut the map into meshes, each with a size of 0.5 [km] * 0.5 [km], and tag the ID for each node (denote node ID as $L$, $L$ ∈ $\left\{ {\left( {0,0} \right), \left( {0, 1} \right),\left( {1,0} \right), ...,\left( {i,j} \right)} \right\}$).

**Step 1-3.** Define $\sigma_{ + x}$ as a function to input a node ID and return the ID of the node that is on the right of that node. Similarly, also define $\sigma_{ + y} , \sigma_{ - x}$ and $\sigma_{ - y}$ (denote the direction mapping set as $\sigma$, $\sigma$ ∈ {$\sigma_{ + x} ,\sigma_{ + y} , \sigma_{ - x} ,\sigma_{ - y}$}).

#### Step 2 (RECM part):

**Step 2-1.** Calculate the population (number of people) $p_{{\left( {d,t,L} \right)}}$ comprising people with non-zero velocities and the instant velocity vector for each person (*i*) $v_{{\left( {d,t,L} \right)}}^{\left( i \right)}$ using the speed and course (angle) variables in the raw GPS data for every node. (If there are no instant speed and course variables in raw data, speed and angle can also be deduced by (user, longitude, latitude, time) log).

**Step 2-2.** Calculate the mean velocity of each person $v_{{\left( {d,t,L} \right)}}$ for each node, where $v_{{\left( {d,t,L} \right)}} = \mathop {{\text{mean}}}\limits_{i} \{ v_{{\left( {d,t,L} \right)}}^{\left( i \right)} \}$. Decompose the vector $v_{{\left( {d,t,L} \right)}}$ into its + *x*, − *x*, + *y*, and -*y* components $v_{{\left( {d,t,L,\sigma_{ + x} } \right)}}$, $v_{{\left( {d,t,L,\sigma_{ - x} } \right)}}$, $v_{{\left( {d,t,L,\sigma_{ + y} } \right)}}$ and $v_{{\left( {d,t,L,\sigma_{ - y} } \right)}}$ respectively.

**Step 2-3.** Calculation of human current: given a node, there are four directions to adjacent nodes, $$I_{{\left( {d,t,L,\sigma_{ + x} } \right)}}$$, $$I_{{\left( {d,t,L,\sigma_{ + y} } \right)}}$$, $$I_{{\left( {d,t,L,\sigma_{ - x} } \right)}}$$, and $$I_{{\left( {d,t,L,\sigma_{ - y} } \right)}}$$, which represent right, up, left, and down currents, respectively. Traverse all the nodes $$L$$, days *d*, and time periods *t*, and calculate the current using Eq. (2) (time complexity: $$O(d*t*L$$)).

**Step 2-4**. Calculation of human resistance: Traverse all the nodes $$L$$, days *d*, and time periods *t* and calculate the resistance using Eq. (4) (time complexity of the new method: $$O(d*t*L$$)).

**Step 2-5.** Calculation of human potential: Calculate the charge $Q_{{\left( {d,t,L} \right)}}$, the divergence of human voltage, at date *d*, time period *t* and location $L$ using the following equation:

9 $$Q_{{\left( {d,t,L} \right)}} = div\left( {I_{d,t,L} *R_{L} } \right) = \mathop \sum \limits_{i = 1}^{4} I_{{\left( {d,t,L,\sigma_{i} } \right)}} *R_{{\left( {L,\sigma } \right)}}$$

The structure of the electrical circuit network on the map is fixed when the observed data are provided. Find the largest connected component of the network using the breadth-first search (BFS) algorithm. The adjacent matrix is defined as follows:

10 $$A_{i,j} = \left\{ {\begin{array}{l} {1,\, node\, i\, is\, adjacent\, to\, j} \\ {0,\, else} \\ \end{array} } \right.$$

Before to define diagonal matrix, sea area and land area need to be defined. According to data downloaded from “Ministry of Land, Infrastructure, Transport and Tourism” Japan, the ratio of sea for each mesh can be calculated, and the sea ratio above 90% of a mesh will be defined as sea area, otherwise it will be defined as land area. Sea nodes will be removed before the calculation, land node adjacent to sea node will be called as sea boundary. Then diagonal matrix for the node on the sea boundary can be defined as follows:

11 $$D_{i,j} = \left\{ {\begin{array}{l} {\mathop \sum \limits_{j} A_{i,j} , case 1} \\ {4, case 2} \\ {0, case 3} \\ \end{array} } \right.$$

where $$case 1:i = j \;and \;node\; i\; is\; adjacent\; to\; the\; sea \;area$$

$$case 2: i = j \; and\; node\; i\; is\; on\; the \;other\; area$$

$$case 3: i \ne j$$

Then, solve the human potential $\emptyset_{d,t,l}$ at day *d*, time period *t*, and location $L$ as follows (More details show in Supplementary Material 4):

12 $$\left( {D_{i,j} - A_{i,j} } \right)*\left( {\begin{array}{*{20}c} {\emptyset_{d,t,1} } \\ \ldots \\ {\emptyset_{d,t,L} } \\ \end{array} } \right) = \left( {\begin{array}{*{20}c} {div\left( {I_{d,t,1} *R_{1} } \right)} \\ \ldots \\ {div\left( {I_{d,t,L} *R_{L} } \right)} \\ \end{array} } \right)$$

Notably, if abnormally large potential value occurs at an abnormal place such as system boundary in rural area where there should not be much people moving, this kind of irregular value must be removed as abnormal value.

#### Step 3 (RGM part):

**Step 3-1**. Determine the boundary of the observation area on the map (shape of the electric circuit network). The shape of the boundary can be arbitrary as long as 1) all the resistors are connected in the network, and 2) the network includes the origin and destination nodes (we have proved that the RGM solution does not rely on the boundary of the system in Supplementary Material 3).

**Step 3-2**. Set a linear equation system $K*I = b$, where $I$ is the current vector to be calculated; $b$ is the vector in which only the last element is the given human voltage 1, and other elements are 0); and $K$ is the coefficient matrix of the linear equation system, which is subject to the following three conditions (Kirchhoff’s law). The number of links is denoted as $N$, and $I$ and $b$ are expressed as follows:

13 $$I = \left( {\begin{array}{*{20}c} {I_{1} } \\ {I_{2} } \\ \vdots \\ {I_{n} } \\ \end{array} } \right)_{N*1}$$

14 $$b = \left( {\begin{array}{*{20}c} 0 \\ \vdots \\ 0 \\ 1 \\ \end{array} } \right)_{N*1}$$

Condition (1) The divergence of the current of all the nodes in the system is equal to 0, except for the origin and destination:

15 $$div\left( {I_{L} } \right) = 0, \quad for\, L \ne origin, destination$$

where $$div\left( {I_{L} } \right) = \mathop \sum \limits_{i = 1}^{4} I_{{\left( {L,\sigma_{i} } \right)}}$$

Zero divergence means the volume flowing in equals to the one flowing out (people will not stay at a node).

Condition (2) The rotation of voltage in the system equals 0:

16 $$rot\left( {I_{L} *R_{L} } \right) = 0, \quad for\, any\, location\, L$$

where $$rot\left( {I_{L} *R_{L} } \right) = \mathop \sum \limits_{k = 0}^{3} I_{{\left( {\mathop \prod \limits_{i = 0}^{k} \sigma_{i} \left( L \right),\sigma_{k + 1} } \right)}} *R_{{\left( {\mathop \prod \limits_{i = 0}^{k} \sigma_{i} \left( L \right),\sigma_{k + 1} } \right)}}$$

$$\sigma_{0} = 1, \sigma_{1} = \sigma_{ + x} , \sigma_{2} = \sigma_{ + y} , \sigma_{3} = \sigma_{ - x} , \sigma_{4} = \sigma_{ - y}$$

This means the following:

$$\begin{aligned} rot\left( {I_{L} *R_{L} } \right) & = I_{{\left( {L,\sigma_{ + x} } \right)}} *R_{{\left( {L,\sigma_{ + x} } \right)}} + I_{{\left( {\sigma_{ + x} \left( L \right),\sigma_{ + y} } \right)}} *R_{{\left( {\sigma_{ + x} \left( L \right),\sigma_{ + y} } \right)}} \\ & \;\;\; - I_{{\left( {L,\sigma_{ + y} } \right)}} *R_{{\left( {L,\sigma_{ + y} } \right)}} - I_{{\left( {\sigma_{ + y} \left( L \right),\sigma_{ + x} } \right)}} *R_{{\left( {\sigma_{ + y} \left( L \right),\sigma_{ + x} } \right)}} \\ \end{aligned}$$

Zero rotation means people will not be wandering around in the same area all the time.

Condition (3) Arbitrarily find one path from the origin to destination such that the summation of the voltage on the path equals 1:

17 $$\mathop \sum \limits_{i \in P} \left( {I_{i} *R_{i} } \right) = 1$$

where link $i$ is on any path $P$ from the origin to destination.

As a simple case, we selected a rectangular observation area with m rows and n column nodes in the system (boundary as shown in Fig. 3a). Overall, there were $N = \left( {m - 1} \right)*n + \left( {n - 1} \right)*m$ links, $M_{1} = m*n$ nodes, and $M_{2} = \left( {m - 1} \right)*\left( {n - 1} \right)$ rotations in the system. These three conditions can be summarized as follows (we set $\sigma_{ + x} and \sigma_{ + y}$ as the positive direction, and $\sigma_{ - x} or \sigma_{ - y}$ as the negative direction):

18 $$K = \left( {\begin{array}{*{20}c} {K_{{1, \left( {M_{1} - 2} \right)*\left( N \right) }} } \\ {K_{{2,\left( {M_{2} } \right)*\left( N \right)}} } \\ {K_{3,\left( 1 \right)*\left( N \right)} } \\ \end{array} } \right)_{N*N}$$

where $$\left( {K_{1} } \right)_{i,j} = \left\{ {\begin{array}{l} {1, \quad if\, link\, j\, is\, connected\, with\, node\, i\, with\, direction\, \sigma_{ + x} or \sigma_{ + y} } \\ { - 1,\quad if\, link\, j\, is\, connected\, with\, node\, i\, with\, direction\, \sigma_{ - x} or \sigma_{ - y} } \\ {0,\,\,else} \\ \end{array} } \right.$$

$$\left( {K_{2} } \right)_{i,j} = \left\{ {\begin{array}{l} {R_{j} ,\quad if\, link\, j\, is\, in\, the\, rotation\, of\, the\, node\, i\, with\, direction\, \sigma_{ + x} or \sigma_{ + y} } \\ { - R_{j} ,\quad if\, link\, j\, is\, in\, the\, rotation\, of\, the\, node\, i\, with\, direction\, \sigma_{ - x} or \sigma_{ - y} } \\ {0,\quad else} \\ \end{array} } \right.$$

$$\left( {K_{3} } \right)_{i,j} = \left\{ {\begin{array}{l} {R_{j} , \quad if\, link\, j\, is\, on\, the\, path\, P\, with\, direction\, \sigma_{ + x} or \sigma_{ + y} } \\ { - R_{j} ,\quad if\, link\, j\, is\, on\, the\, path\, P\, with\, direction\, \sigma_{ - x} or \sigma_{ - y} } \\ {0,\quad else} \\ \end{array} } \right.$$

Since $\left( {M_{1} - 2} \right) + \left( {M_{2} } \right) + \left( 1 \right) = N$, and each condition is independent^48^, the coefficient matrix is full-rank, meaning that there is a unique solution to the linear equation system according to linear algebra.

**Step 3-3**. Learn the human resistance value from the real data by using the method in RECM, solve the linear equations in step 3**-**2 to generate the human current, and plot it on the map to observe the manner in which people flow from the origin to destination.

**Step 3-4 (Optional)**. If humans cannot pass through some node for some reason, set up an “accident-affected area” in the map, that is, set the value of resistance on the link around the accident-affected area to infinity (a large number in numerical calculation) to simulate the human flow when accident area occurs.

### Time complexity of the algorithms

Regarding to time complexity of the algorithms, the time complexity in calculating the resistance was $$O\left( {iter*d*t*L} \right)$$ in the previous study, whereas that in our study was $$O(d*t*L$$) after our improvement. In Tokyo, the number of nodes, iterations, days, and time periods were approximately 10^4^, 10^4^, 10^2^, and 10^1^, respectively. Therefore, the time cost of the number of nodes and iterations was dominant, and the computational cost was reduced to 1/10,000 of the original cost. In a real-world scenario, it took approximately 1–2 h to calculate the current, 3–5 days (using the Adam algorithm^49^ before, but now only 1 h) to calculate the resistance, and 1–2 h to calculate the electric potential for the Greater Tokyo Area, which had approximately 30,000 nodes (node size: 500 m * 500 m; computational environment shown in Method). Using the RECM, the same result was obtained at a computational cost that was drastically decreased, indicating that we made it easier to deploy and more useful for other researchers. To use the RGM, a linear equation system needs to be solved. Its theoretical time complexity is $$O\left( {n^{3} } \right)$$, where $$n$$ is the number of nodes in the system. Since its coefficient matrix is sparse, the method proposed by Peng et al.^50^ for solving the linear equation can be utilized to practically reduce the time complexity cost to $$O\left( {n^{2.33} } \right)$$.

### Supplementary Information

Supplementary Information.

## Data Availability

Data used in this study can be purchased from a Japanese private company, Agoop, which sells "The location information big data which acquired from the smart phone app". The product name in Japanese is "Pointo-gata ryudou-jinkou data" ("Point-type population data").

## References

[1] Schläpfer M (2021). The universal visitation law of human mobility. Nature.

[2] Kantor P (1987). The dependent city: the changing political economy of urban economic development in the United States. Urban Affairs Q..

[3] Anas A, Arnott R, Small KA (1998). Urban spatial structure. J. Econ. Literat..

[4] Cuttone A, Lehmann S, González MC (2018). Understanding predictability and exploration in human mobility. EPJ Data Sci..

[5] Rickwood P, Glazebrook G, Searle G (2008). Urban structure and energy—a review. Urban Policy Res..

[6] Mahutga MC, Ma X, Smith DA, Timberlake M (2010). Economic globalization and the structure of the world city system: the case of airline passenger data. Urban Stud..

[7] Shida Y, Takayasu H, Havlin S, Takayasu M (2020). Universal scaling laws of collective human flow patterns in urban regions. Sci. Rep..

[8] Bonaccorsi G (2020). Economic and social consequences of human mobility restrictions under COVID-19. Proc. Natl. Acad. Sci..

[9] Bonaccorsi, G. *et al*. Economic and social consequences of human mobility restrictions under COVID-19. In *Proceedings of the National Academy of Sciences, vol. 117* 15530–15535 (2020). 10.1073/pnas.2007658117 PMC7355033 32554604

[10] Yabe T, Zhang Y, Ukkusuri SV (2020). Quantifying the economic impact of disasters on businesses using human mobility data: a Bayesian causal inference approach. EPJ Data Sci..

[11] Bergstrand JH (1985). The gravity equation in international trade: Some microeconomic foundations and empirical evidence. Rev. Econ. Stat..

[12] Carra G, Mulalic I, Fosgerau M, Barthelemy M (2016). Modelling the relation between income and commuting distance. J. R. Soc. Interface.

[13] Mohammadi N, Taylor JE (2017). Urban infrastructure-mobility energy flux. Energy.

[14] Jiang B, Yin J, Zhao S (2009). Characterizing the human mobility pattern in a large street network. Phys. Rev. E.

[15] Chang S (2021). Mobility network models of COVID-19 explain inequities and inform reopening. Nature.

[16] Ozaki J, Shida Y, Takayasu H, Takayasu M (2022). Direct modelling from GPS data reveals daily-activity-dependency of effective reproduction number in COVID-19 pandemic. Sci. Rep..

[17] Batty M (1971). Modelling cities as dynamic systems. Nature.

[18] Philbrik AT (1973). A short history of the development of the gravity model.". Austral. Road Res..

[19] Ewing GO (1974). Gravity and linear regression models of spatial interaction: A cautionary note. Econ. Geogr..

[20] Erlander, S. & Stewart, N. F. *The Gravity Model in Transportation Analysis: Theory and Extensions* (Springer, 1990).

[21] Karemera D, Oguledo VI, Davis B (2000). A gravity model analysis of international migration to North America. Appl. Econ..

[22] Jung W-S, Wang F, Stanley HE (2008). Gravity model in the Korean highway. Europhys. Lett..

[23] Song C, Koren T, Wang P, Barabási A-L (2010). Modelling the scaling properties of human mobility. Nat. Phys..

[24] Gonzalez MC, Hidalgo CA, Barabasi A-L (2008). Understanding individual human mobility patterns. Nature.

[25] Song C, Zehui Q, Blumm N, Barabási A-L (2010). Limits of predictability in human mobility. Science.

[26] Chen L, Lv M, Ye Q, Chen G, Woodward J (2011). A personal route prediction system based on trajectory data mining. Inf. Sci..

[27] Lu X, Wetter E, Bharti N, Tatem AJ, Bengtsson L (2013). Approaching the limit of predictability in human mobility. Sci. Rep..

[28] Toch E, Lerner B, Ben-Zion E, Ben-Gal I (2019). Analyzing large-scale human mobility data: A survey of machine learning methods and applications. Knowl. Inf. Syst..

[29] Jia W, Zhao S, Zhao K (2023). Human mobility prediction based on trend iteration of spectral clustering. IEEE Trans. Mobile Comput..

[30] Dang, W. *et al*. Predicting human mobility via graph convolutional dual-attentive networks. In *Proceedings of the Fifteenth ACM International Conference on Web Search and Data Mining* 192–200 (2022).

[31] Xia F, Wang J, Kong X, Wang Z, Li J, Liu C (2018). Exploring human mobility patterns in urban scenarios: A trajectory data perspective. IEEE Commun. Mag..

[32] Mazzoli M, Molas A, Bassolas A, Lenormand M, Colet P, Ramasco JJ (2019). Field theory for recurrent mobility. Nat. Commun..

[33] Aoki T, Fujishima S, Fujiwara N (2022). Urban spatial structures from human flow by Hodge-Kodaira decomposition. Sci. Rep..

[34] Aoki T, Fujiwara N, Fricker M, Nakagaki T (2022). A model for simulating emergent patterns of cities and roads on real-world landscapes. Sci. Rep..

[35] Shida Y, Ozaki J, Takayasu H, Takayasu M (2022). Potential fields and fluctuation-dissipation relations derived from human flow in urban areas modeled by a network of electric circuits. Sci. Rep..

[36] Ďuriš V, Chertanovskiy AG, Chumarov SG, Kartuzov AV (2022). Calculation of electric circuits using the Fast Kirchhoff method. TEM J..

[37] Magzhan K, Jani HM (2013). A review and evaluations of shortest path algorithms. Int. J. Sci. Technol. Res..

[38] Yu M, Yang C, Li Y (2018). Big data in natural disaster management: A review. Geosciences.

[39] Song X (2016). Prediction and simulation of human mobility following natural disasters. ACM Trans. Intell. Syst. Technol. (TIST).

[40] Song, X. *et al*. A simulator of human emergency mobility following disasters: Knowledge transfer from big disaster data. In *Proceedings of the AAAI Conference on Artificial Intelligence, vol. 29* 1 (2015).

[41] Stute, M., Maass, M., Schons, T. & Hollick, M. Reverse engineering human mobility in large-scale natural disasters. In *Proceedings of the 20th ACM International Conference on Modelling, Analysis and Simulation of Wireless and Mobile Systems* 219–226 (2017).

[42] Wang Q, Taylor JE (2016). Process map for urban-human mobility and civil infrastructure data collection using geosocial networking platforms. J. Comput. Civ. Eng..

[43] Merris R (1994). Laplacian matrices of graphs: A survey. Linear algebra Appl..

[44] Bapat RB (1996). The Laplacian matrix of a graph. Math. Student-India.

[45] Anderson J, William N, Morley TD (1985). Eigenvalues of the Laplacian of a graph. Linear Multilinear Algebra.

[46] Gutman I, Xia W (2004). Generalized inverse of the Laplacian matrix and some applications. Bulletin.

[47] Bozzo E (2013). The Moore-Penrose inverse of the normalized graph Laplacian. Linear Algebra Appl..

[48] Feldmann P, Rohrer RA (1991). Proof of the number of independent Kirchhoff equations in an electrical circuit. IEEE Trans. Circ. Syst..

[49] Kingma, D. P. & Jimmy, B. Adam: A method for stochastic optimization. arXiv:1412.6980 (2014).

[50] Peng, R. & Santosh, V. Solving sparse linear systems faster than matrix multiplication. In *Proceedings of the 2021 ACM-SIAM Symposium on Discrete Algorithms (SODA)* 504–521 (Society for Industrial and Applied Mathematics, 2021).

[51] Fukuda Y (2020). Land prices and agglomeration: Theory and evidence from the Tokyo metropolitan area. J. Jpn. Int. Econ..

[52] McDonald JF, Prather PJ (1994). Suburban employment centres: The case of Chicago. Urban Stud..

[53] Siedentop S, Fina S, Krehl A (2016). Greenbelts in Germany's regional plans—an effective growth management policy?. Landsc. Urban Plan..

[54] Zhang X, Xu Y, Tu W, Ratti C (2018). Do different datasets tell the same story about urban mobility—a comparative study of public transit and taxi usage. J. Transport Geogr..

[55] Zhong C, Schläpfer M, Arisona SM, Batty M, Ratti C, Schmitt G (2017). Revealing centrality in the spatial structure of cities from human activity patterns. Urban Stud..

[56] Taubenböck H, Standfuß I, Wurm M, Krehl A, Siedentop S (2017). Measuring morphological polycentricity-A comparative analysis of urban mass concentrations using remote sensing data. Comput. Env. Urban Syst..

[57] Fujita M, Ogawa H (1982). Multiple equilibria and structural transition of non-monocentric urban configurations. Region. Sci. Urban Econ..

[58] Barthelemy M (2019). The statistical physics of cities. Nat. Rev. Phys..

[59] Huang Z, Wang P, Zhang F, Gao J, Schich M (2018). A mobility network approach to identify and anticipate large crowd gatherings. Transport. Res. Part B: Methodol..

[60] Fan, Z., Xuan, S., Ryosuke, S. & Ryutaro, A. Citymomentum: an online approach for crowd behavior prediction at a citywide level. In *Proceedings of the 2015 ACM International Joint Conference on Pervasive and Ubiquitous Computing* 559–569 (2015).

[61] Calabrese F, Mi D, Di Giusy L, Joseph F, Carlo R (2013). Understanding individual mobility patterns from urban sensing data: A mobile phone trace example. Transport. Res. Part C: Emerg. Technol..

