# Supplementary material for: Human mobility description by physical analogy of electric circuit network based on GPS data
Source: Sci Rep. 2024 Jun 11;14:13380. doi: 10.1038/s41598-024-63719-z (PMC11167031; doi:10.1038/s41598-024-63719-z)
Supplement: Supplementary file 1 — Supplementary Information. [file 41598_2024_63719_MOESM1_ESM.docx]

**Human mobility description by physical analogy of electric circuit network based on GPS data**

**Supplementary Material**

**Authors:** Zhihua Zhong^1^, Hideki Tayakasu^1,2^, Misako Takayasu^1^

**Institutions and Affiliations:**

^1^ *School of Computing, Tokyo Institute of Technology, Tokyo, Japan*.

^2^ *Sony Computer Science Laboratories, Tokyo, Japan*.

**Correspondence Author:**

Misako Takayasu

School of Computing, Tokyo Institute of Technology, 2-12-1 Ookayama, Meguro-ku, Tokyo, Japan, 152-8550

Email: takayasu.m.aa@m.titech.ac.jp

Contents

[**1. Brief introduction** 1](#_Toc164155965)

[**2. Discussion about new and old resistance and rotation** 1](#_Toc164155966)

[**2.1 Review of the method to calculate resistance in the previous research** 2](#_Toc164155967)

[**2.2 Limitation of the old method** 3](#_Toc164155968)

[**2.3 New methods to calculate the resistance** 4](#_Toc164155969)

[**2.4 Current is subject to exponential distribution** 7](#_Toc164155970)

[**2.5 Comparison of new and old resistance and rotation** 8](#_Toc164155971)

[**2.6 Conductivity spatial distribution on different cities in Japan** 13](#_Toc164155972)

[**3. Route Generation Model** 14](#_Toc164155973)

[**3.1 The method we recommend using to determine resistance** 15](#_Toc164155974)

[**3.2 Boundary selection of RGM** 16](#_Toc164155975)

[**3.3 More examples of route generation** 17](#_Toc164155976)

[**4. Review of the method of calculating human potential** 18](#_Toc164155977)

[**5. Brief introduction of our open-source code** 19](#_Toc164155978)

[**Reference:** 20](#_Toc164155979)

# **1. Brief introduction**

In supplementary, detailed content which could not be included in the main part due to words limitation will be discussed. In section 2, we will discuss the difference between new (in our research) and old (in previous research^1^) resistance and rotation, showing that even we changed the definition of resistance, results in the previous research still can be reproduced. In section 3.1, we recommend our new method (using the mean value of current to calculate resistance) and explain its three advantages. We show that the solution of RGM (route generation model) does not rely on the system's boundary, and people can choose the observation area they are interested in by generating a route between the origin and destination in 3.2. More examples of the route generated by RGM will be shown in 3.3. Moreover, the detailed method of calculating temporal human potential was not written clearly in the previous paper, and we will complement it as a supplement to the former research in section 4. Finally, the place our open-source code release and the tutorial to use our code will be introduced in section 5.

**Raw GPS data examples:**

Examples of raw GPS data are shown in Fig. 1. Researchers with GPS data including time, longitude, latitude, speed, and course (angle) can conduct research about human flow pattern by using our model.


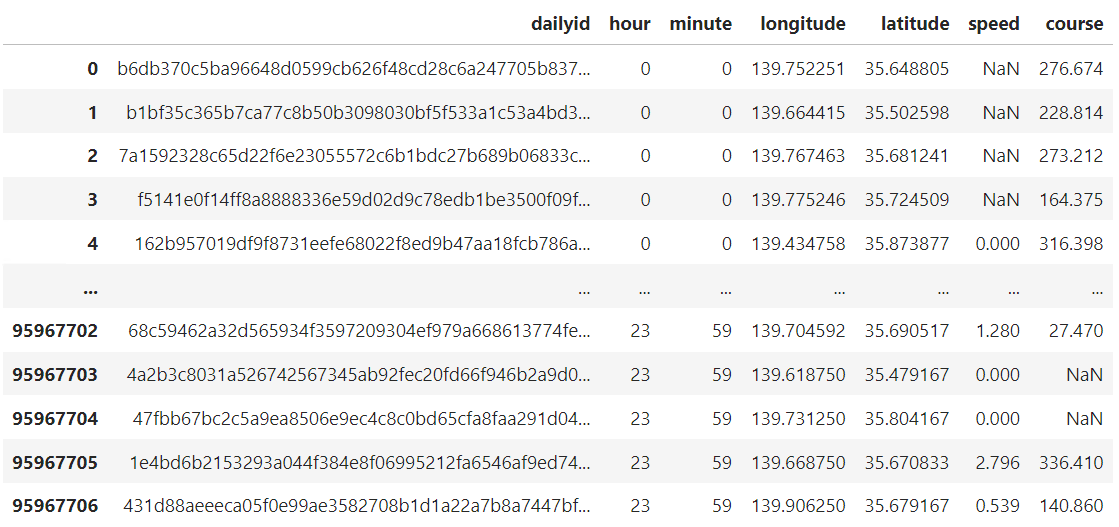


Figure 1. example of raw GPS data (Note: speed and course are not necessary for raw data because it can be calculated if (user, time, longitude, latitude) are known)

# **2. Discussion about new and old resistance and rotation**

In order to show that our new method can replace the old method to calculate resistance in the previous research, in this section, the property of resistance will be discussed in detail. We will introduce the method to calculate resistance in the previous research in 2.1, explain four limitations of the method proposed in the previous research in 2.2, describe our idea of new methods to calculate resistance in detail in 2.3, show that human current is subject to exponential distribution in 2.4, compare the new and old resistance and rotation in 2.5, show the conductivity spatial distribution in different Japanese cities in 2.6.

# **2.1 Review of the method to calculate resistance in the previous research**

In the previous research^1^, the authors calculated resistance by the following inspiration: in physics, there should be no rotation in electric field of an electric circuit with stationary current. They defined the rotation at day *d*, time *t*, and location $L$ by the following formula:

$$rot\left( I_{d,t,L}*R_{L} \right)=I_{\left( d,t,L,\sigma_{+x} \right)}*R_{\left( L,\sigma_{+x} \right)}+I_{\left( d,t,\sigma_{+x}\left( L \right),\sigma_{+y} \right)}*R_{\left( \sigma_{+x}\left( L \right),\sigma_{+y} \right)}-$$

$$I_{\left( d,t,L,\sigma_{+y} \right)}*R_{\left( L,\sigma_{+y} \right)}-I_{(d,t,\sigma_{+y}(L),\sigma_{+x})}*R_{(\sigma_{+y}(L),\sigma_{+x})}$$

For simplicity in notation this equation is written as:

$rot\left( I_{d,t,L}*R_{L} \right)=\sum_{k=0}^{3} I_{(d,t,\prod_{i=0}^{k} \sigma_{i}(L),\sigma_{i+1)}}*R_{(\prod_{i=0}^{k} \sigma_{i}(L),\sigma_{i+1})}$ (1)

where, $\sigma_{0}=1:\left( x,y \right)\to\left( x,y \right),\sigma_{1}=\sigma_{+x}:\left( x,y \right)\to\left( x+1,y \right),\sigma_{2}=\sigma_{+y}:\left( x,y \right)\to\left( x,y+1 \right),\sigma_{3}=\sigma_{-x}:(x,y)\to(x-1,y), \sigma_{4}=\sigma_{-y}:(x,y)\to(x,y-1)$

They calculated the resistance value by treating it as an optimization problem whose objective function is the square of the summation of the rotation of nodes around the map, as shown in the following formula. The optimization problem is subject to, firstly that $R_{(l,\sigma)}$, resistance value at location $L$ and direction $\sigma$, should be greater than 0, and secondly they assume a kind of conservation rule that $\sum_{L, \sigma} R_{(L,\sigma)}^{\left( 0 \right)}=\sum_{L, \sigma} R_{(L,\sigma)}^{\left( \tau\right)}$($\tau^{th}$ iteration's summation of resistance equal to $1^{st}$ iteration's summation of resistance).

$minimize L=\sum_{d,t,L} \left( rot\left( I_{d,t,L}*R_{L} \right) \right)^{2}-\gamma*\sum_{(L,\sigma)} Log\left( R_{(L,\sigma)} \right)$(2)

$$subject to: \sum_{L, \sigma} R_{\left( L,\sigma\right)}^{\left( 0 \right)}=\sum_{L, \sigma} R_{\left( L,\sigma\right)}^{\left( \tau\right)}$$

where, $R_{(L,\sigma)}^{\left( 0 \right)}=1$ for each location $L$ and direction $\sigma$

Adam^4^, an optimization algorithm popular in deep learning, was employed to solve this optimization problem to get the values of resistance. Adam will update resistance by the following form:

$R_{(L,\sigma)}^{\left( \tau\right)}=R_{(L,\sigma)}^{\left( \tau-1 \right)}-\alpha*f(\frac{\partial L}{\partial R_{(L,\sigma)}^{\left( \tau-1 \right)}})$ (3)

where $\alpha$ is learning rate

$\frac{\partial L}{\partial R_{L}}=(2*I_{d,t,L}*\sum_{d,t} rot\left( I_{d,t,L}*R_{L} \right)-\gamma*\frac{1}{R_{L}}$ (4)

There are cases, $R_{(L,\sigma)}^{\left( \tau\right)}$ drops below 0 after an update, if so, authors adjusted the parameter $\gamma$ to $10*\gamma$, ${10}^{2}*\gamma$, ${10}^{3}*\gamma$, …, until the value of $R_{(L,\sigma)}^{\left( \tau\right)}$ becomes positive once again. Meanwhile, to keep $\sum_{L, \sigma} R_{(L,\sigma)}^{\left( 0 \right)}=\sum_{L, \sigma} R_{(L,\sigma)}^{\left( \tau\right)}$, authors applied a normalization after each iteration by the following formula (5):

$R_{(L,\sigma)}^{\left( \tau\right)}:=R_{(L,\sigma)}^{\left( \tau\right)}*(\frac{\sum_{L,\sigma} R_{(L,\sigma)}^{\left( \tau-1 \right)}}{\sum_{L,\sigma} R_{(L,\sigma)}^{\left( \tau\right)}})$ (5)

# **2.2 Limitation of the old method**

There are mainly four limitations to calculating resistance using the method suggested in the previous research.

The first problem is the high computational cost of calculating resistance. Our computational environment is CPU: 2 * Intel Xeon Gold 6248R (24 core); Memory: 500 GB; OS: Linux Ubuntu. In real-time, it took about 1-2 hours to calculate current, 3-5 days to calculate resistance, and 1-2 hours to calculate the electric potential for the greater Tokyo area, with about 30000 nodes (node size: 500m * 500m) in the real world time. The computational cost problem of human resistance need to be solved, otherwise the application of ECM will be limited.

The second problem is the difficulty in fine-tuning the hyperparameter of Adam. When using ECM on a new city's data, there is no standard way to find the best parameter, therefore trying different combinations of parameters and waiting a long time before checking whether it works is the only thing can do, which means the old model is not easy to be deployed and used.

The third problem is that the calculated value of resistance depends on the initial value and hyperparameter of Adam. Even though people manage to make Adam converge, resistance value will not be unique. Suppose researchers have two cities' data, such as Tokyo's and New York's. In that case, their suitable hyperparameter may differ, and directly comparing the value of resistance calculated between two cities is meaningless. Moreover, because the electric potential calculation is based on the value of resistance, comparing the potential value between different cities is also meaningless. Above problems can be solved by our proposed new method. Therefore, it is interesting to conduct further research, like comparing the difference of human electric potential between different cities.

The fourth problem is that the method in the previous research will cause some outliers (calculation errors) when calculating resistance for rural areas. Fig. 2 shows the histogram of resistance calculated by our method and the old method on the left and the right, respectively. There is a peak on the far right of the histogram of old resistance. It is because the current value is very small in rural areas, which account for 70% of the greater Tokyo area. In Eq. (3-4), when the current is small, $\frac{\partial L}{\partial R_{L}}$ is also tiny, making $R_{(L,\sigma)}^{\left( \tau\right)}$ cannot update, and the value of $R_{(L,\sigma)}^{\left( \tau\right)}$ always equals the initial value of 1. However, in Eq.(5), the normalization term value $\frac{\sum_{L,\sigma} R_{(L,\sigma)}^{\left( \tau-1 \right)}}{\sum_{L,\sigma} R_{(L,\sigma)}^{\left( \tau\right)}}$ is slightly greater than one because after each time updated, the resistance value $\sum_{L,\sigma} R_{(L,\sigma)}^{\left( \tau\right)}$ will become smaller. Therefore, after 10,000 iterations, resistance in the rural area is approximately equal to 1*$\left( \frac{\sum_{L,\sigma} R_{(L,\sigma)}^{\left( \tau-1 \right)}}{\sum_{L,\sigma} R_{(L,\sigma)}^{\left( \tau\right)}} \right)^{10000}\approx2-3$. These rural areas occupy a large amount of ratio in Tokyo, and it is the reason for the appearance of the peak (calculation error) on the right of Fig. 2. However, the calculation of human potential mainly relies on the current value in the nodes near the city centre, not the rural area. Therefore, human potential can still be correctly calculated in the previous research.


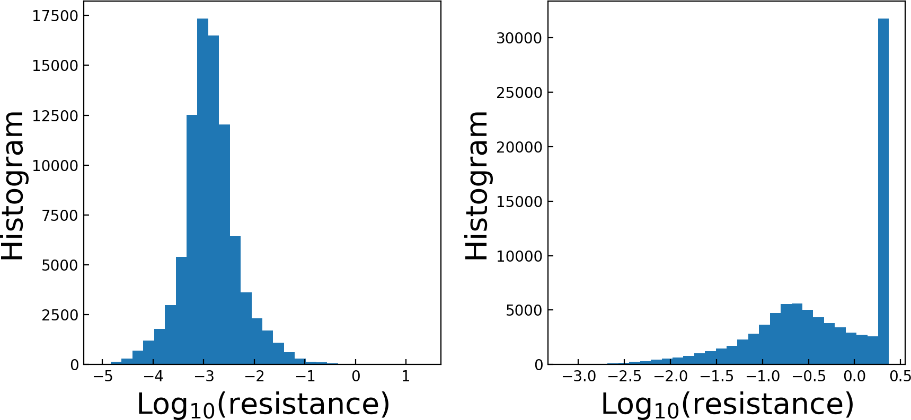


Figure 2. Histogram of new and old resistance

(left: method in our research, right: method in previous research)

# **2.3 New methods to calculate the resistance**

To improve ECM, we paid attention to a relation reported in the previous research, that human conductivity, as the inverse of resistance $\rho_{(L,\sigma)}$, at location $L$, direction $\sigma$ is proportional to the maximum absolute current value $\max_{d,t} \left\{ \left| I_{(d,t,L,\sigma)} \right| \right\}$ at location $L$, direction $\sigma$. That is:

$\rho_{(L,\sigma)}\propto\max_{d,t} \left\{ \left| I_{\left( d,t,L,\sigma\right)} \right| \right\}$ (6)

Because conductivity is the inverse of resistance, resistance value equals a constant k divided by the maximum absolute current value. Let's assume simply that constant k equals 1. Then resistance can be calculated by the inverse of its maximum current. (Method 1)

$R_{(L,\sigma)}=\frac{1}{{max}_{d,t} \left\{ \left| I_{(d,t,L,\sigma)} \right| \right\}}$ (7)

Based on the resistance calculated by Method 1, temporal human potential can be calculated and compared with the one calculated in previous research. Fig. 4 shows a robust linear relationship between new and old results. R square of linear regression is 0.95, which means people can use the potential value calculated by our new method to replace the old one.

We tried to plot the CDF (cumulative density function) of absolute value current in many famous locations in Tokyo, such as Kourakuen, Kazo, Hakone, and Tokyo station as shown in Fig. 3. CDF shows that the current distribution in these nodes approximated by the exponential distribution. However, fluctuation of the maximum value is generally very large, so using the maximum current to determine resistance may not be robust.


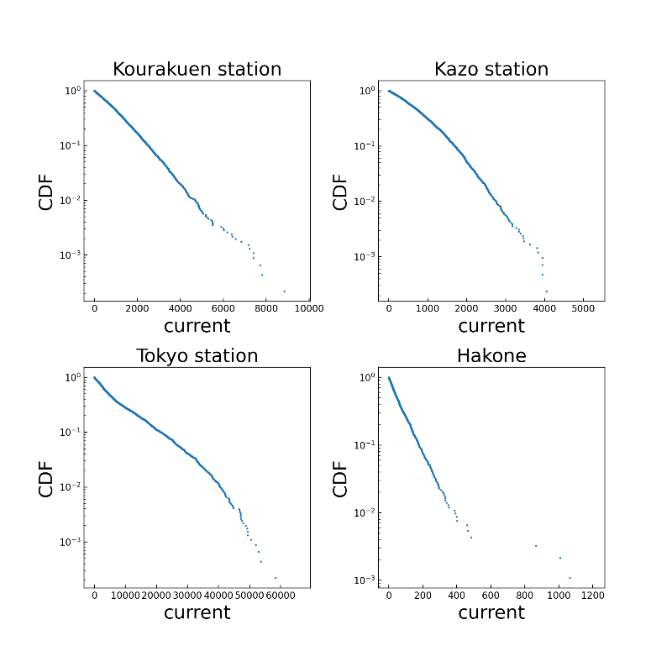


Figure 3. CDF of current in different places

In order to find a better definition, we searched the cases of 97.5% quantile (Method 2), 95% quantile current (Method 3), and the parameter of exponential distribution of current (the mean absolute current, Method 4) to determine resistance.

$R_{(L,\sigma)}=\frac{1}{{Quantile}_{0.975, d,t} \left\{ \left| I_{(d,t,L,\sigma)} \right| \right\}}$ (8)

$R_{(L,\sigma)}=\frac{1}{{Quantile}_{0.95,d,t} \left\{ \left| I_{(d,t,L,\sigma)} \right| \right\}}$ (9)

Fig. 4 shows the result of the relationship between old potential and new potential calculated by different methods. When using 97.5% quantile and 95% quantile current to calculate resistance, R square of linear regression improved, which confirms our conjecture that maximum current is not robust.


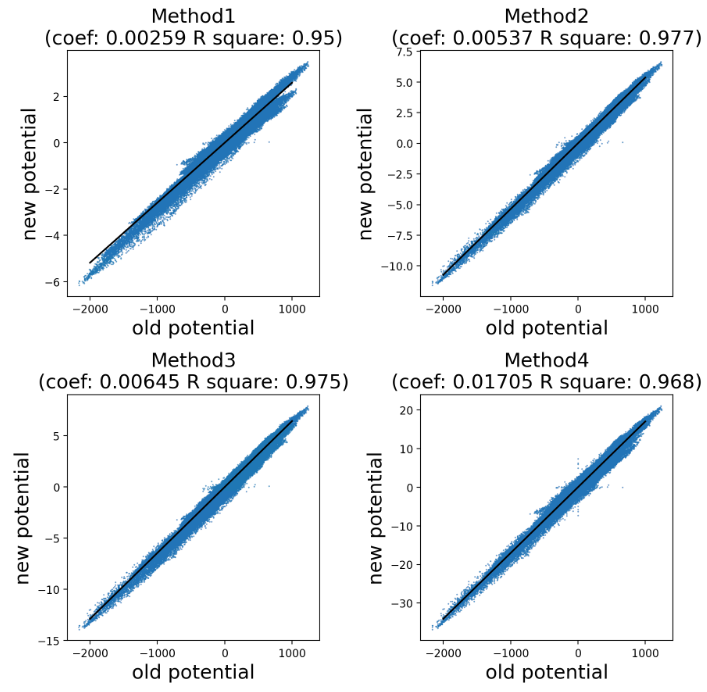


Figure 4. Comparison of potential calculated by the new and old method

(Note: method 1: maximum current, method 2: 97.5% quantile current,

method 3: 95% quantile current, method 4: mean current)

Time series for a different day of different per cent quantile current on a certain day was plotted in Tokyo Station (left: area with high population) and Hakone (right: area with low population), as shown in Fig. 5. We denote current time series at a specific quantile *q*, at day *d*, at location $L$ as:

${CTS}_{(q,d,L)}{=Quantile}_{q,t} \{\left| I_{(d,t,L)} \right|\}$(10)

In Fig. 5, we observe that the current series ${CTS}_{q,d,L=Tokyo Station}$ fluctuate strongly when quantile *q* is high (*q*∈[0,1]). For example, we pay attention to the blue line (maximum current of every day) in Tokyo station, ${CTS}_{(q=1,d,L=Tokyo station)}$. If the observation period is $d_{1}=[1, 10]$ or $d_{2}=[11, 20]$, the difference between the value of the right current (blue line) gained in $d_{1}$ and $d_{2}$ is large (12500 vs 17500). The resistance value is expected to be independ of the observation period because resistance should be a quantity reflecting the infrastructure level of a place independent of time. Therefore, it is better to use a per cent quantile *q* that the standard deviation of ${CTS}_{q,d,L}$ is small, which means that no matter which time people observe, the resistance value will not change too much.


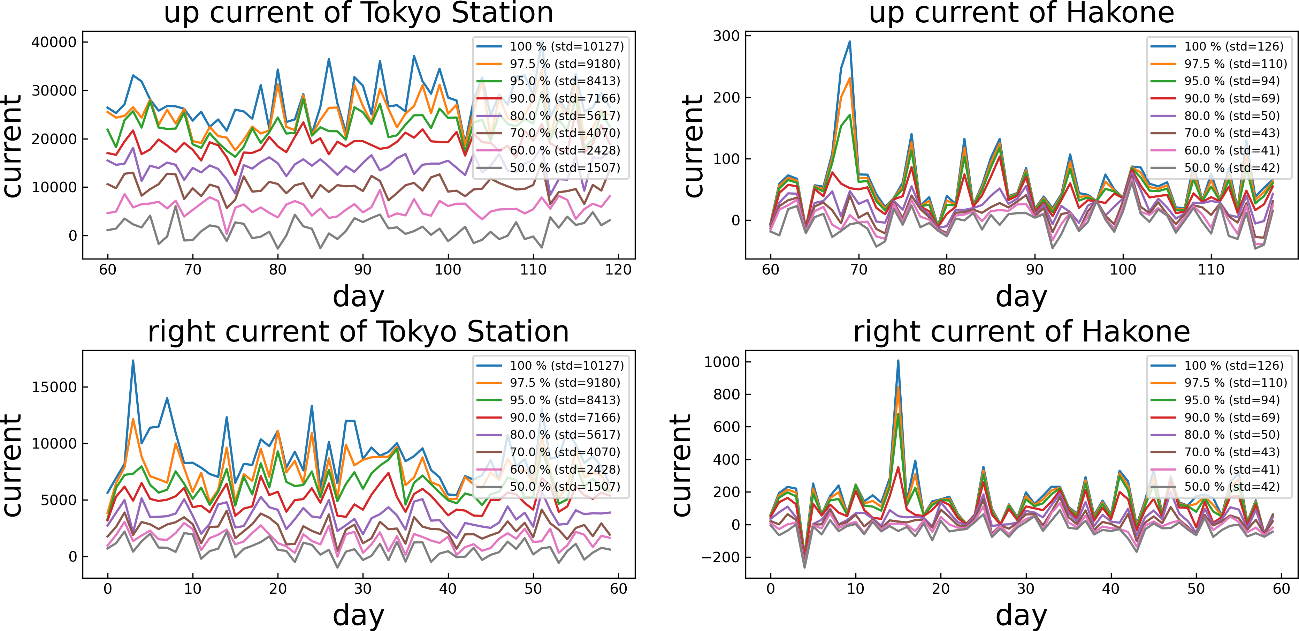


Figure 5. Time series of current at different places (left: Tokyo Station, right: Hakone)

Relation between the quantile and standard deviation of ${CTS}_{q,d,L}$ was plotted in Fig. 6. When the quantile decreased, the standard deviation dropped rapidly at the beginning and became steadier later, which inspired us to use Elbow Method to determine the suitable quantile. For example, the shape of the curve looks like the human arm, before 95%, standard deviation changes rapidly, and after that, it gradually converges. Therefore, we can use a percentage of 95% or 92.5% to determine resistance.


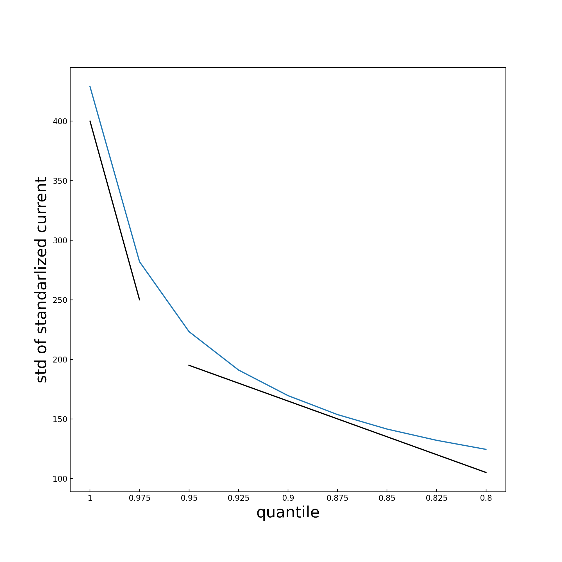


Figure 6. Relationship between quantile and standard deviation of ${CTS}_{q,d,L}$

# **2.4 Current is subject to exponential distribution**

As we mentioned before, in Fig. 3, we noticed a phenomenon: current distributions in many places are close to the exponential distribution. Therefore, K-S test was employed to check whether the current is subject to exponential distribution in most places in the greater Tokyo area. In Fig. 7, areas where the p-value of the K-S test is greater than 0.05, are shown in blue, which means that in most places, human current distribution can be treated as exponential distribution.


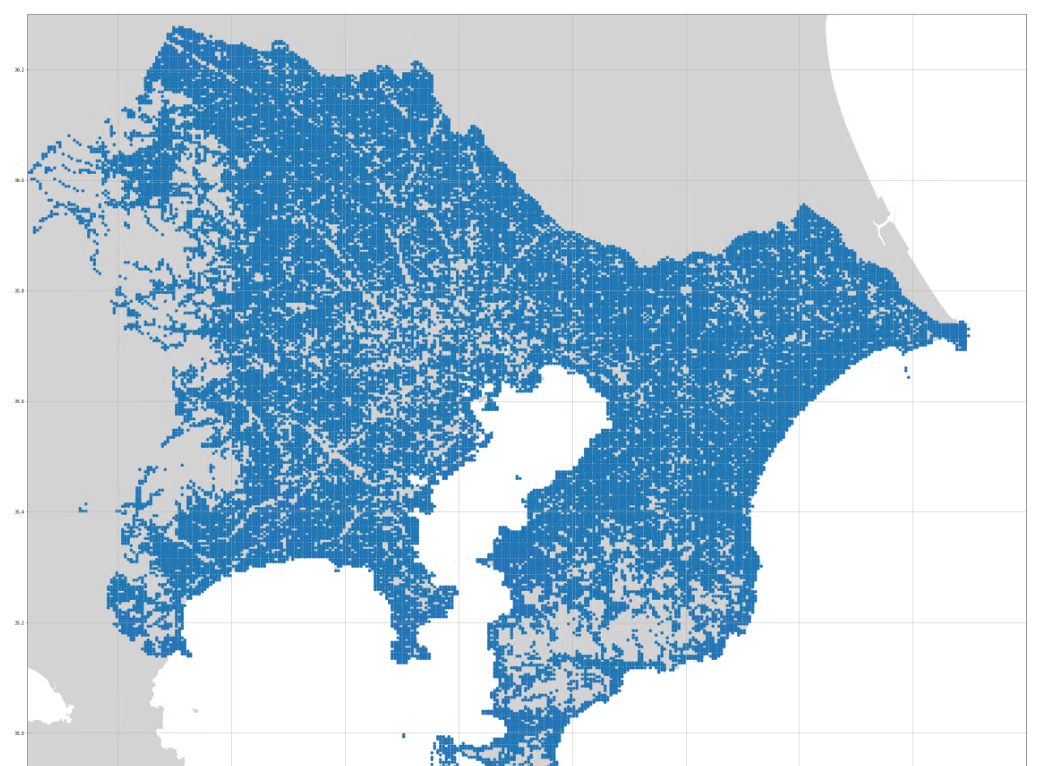


Figure 7. KS-test result in the greater Tokyo area (blue area: p-value is greater than 0.05)

Therefore, we write the PDF (Probability density function) of the current for each location $L$ and direction $\sigma$ in the following form:

$p\left( I_{(L,\sigma)} \right)\sim\left( \frac{1}{I_{(L,\sigma)}^{0}} \right)*e^{-\frac{\left| I_{(d,t,L,\sigma)} \right|}{I_{(L,\sigma)}^{0}}}$ (11)

where $I_{(L,\sigma)}^{0}=\underset{d,t}{mean} \{\left| I_{(d,t,L,\sigma)} \right|\}$

Being the parameter of the exponential distribution, $I_{L,\sigma}^{0}$ means the mean current at location $L$, direction $\sigma$. It gave us a hint that we may also use $I_{L,\sigma}^{0}$ to determine resistance as follows (Method 4):

$R_{(L,\sigma)}=\frac{1}{I_{(L,\sigma)}^{0}}=\frac{1}{\underset{d,t}{mean} \{|I_{(d,t,L,\sigma)}|\}}$ (12)

As shown in Fig. 4, there is little difference between new and old potential concerning the different proposed methods. Furthermore, we will show there are not much difference in resistance and rotation values no matter which new methods are used in the following section 2.5 by comparing new and old resistance and rotation.

# **2.5 Comparison of new and old resistance and rotation**

Fig. (8-9) shows the CDF of resistance and conductivity (infrastructure level) calculated by different methods. When quantile *q* decrease, the value of the current time series ${CTS}_{(q,d,L)}$ will decrease, the resistance value increases and the conductivity value decreases.


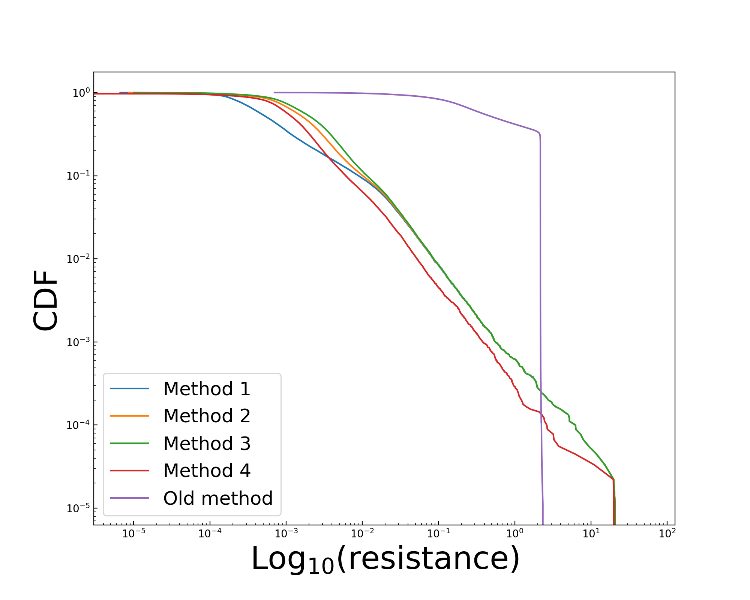


Figure 8. Comparison of CDF of resistance calculated in different methods

(Note: method 1: maximum current, method 2: 97.5% quantile current,

method 3: 95% quantile current, method 4: mean current)


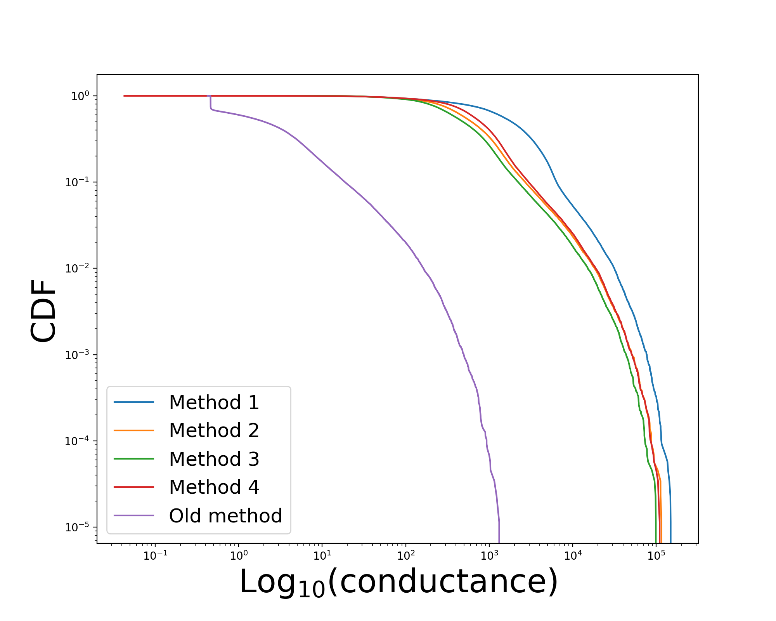


Figure 9. Comparison of CDF of conductivity calculated in different methods

(conductivity is the inverse number of resistance, meaning the infrastructure level)

Fig. 10 shows a linear relationship between new and old resistance. The fluctuation on the far right is caused by the outlier (calculation error) we mentioned in 2.2, Fig. 2. The calculation error problem has also been solved in our new methods.


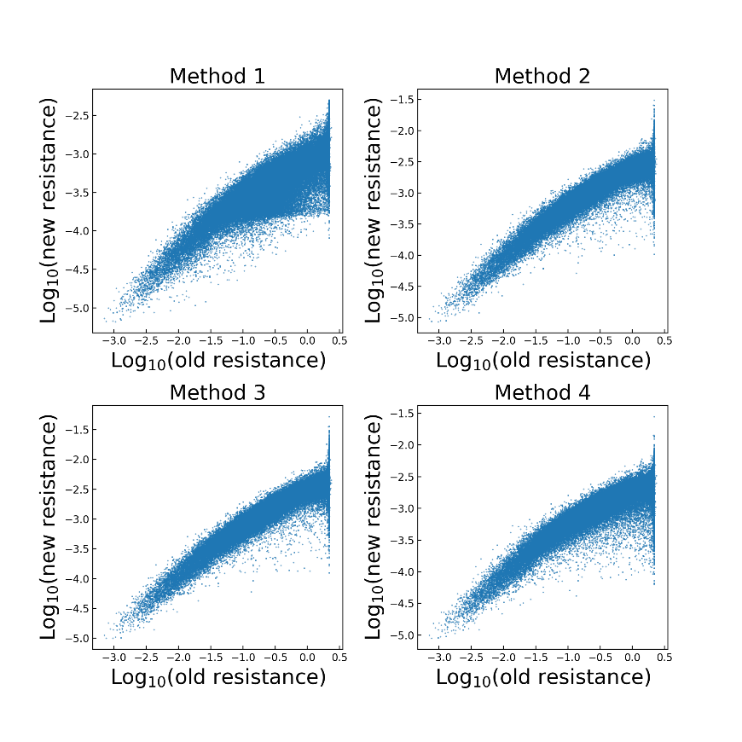


Figure 10. The relationship between old and new resistance concerning different methods (Note: method 1: maximum current, method 2: 97.5% quantile current,

method 3: 95% quantile current, method 4: mean current)

In the previous research, authors tried to reduce rotation of electric field in the electric circuit, and authors calculated resistance by treating it as an optimization problem minimizing the sum of squared rotation in the former method.While, our new method does not need to consider rotation when calculating resistance. Therefore, a problem naturally emerged regarding the rotation in our case. We calculated rotation and compared the value of the new rotation with the old one. Interestingly, Fig.11 shows a linear relationship between the new and old rotation, which shows that the small value in the old rotation is also small in the new one, and the large value in the old rotation is also large in the new one.


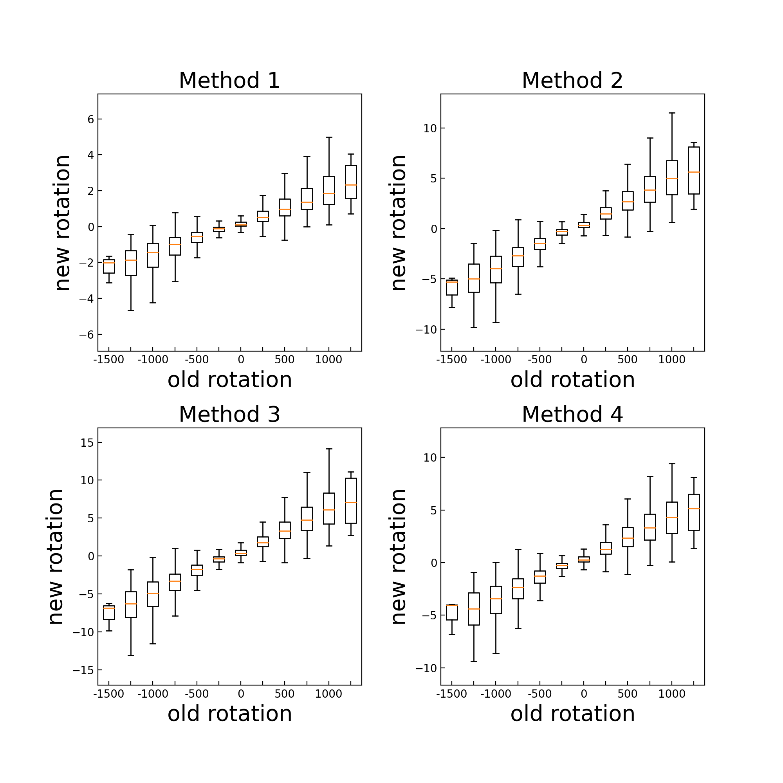


Figure 11. The relationship between old and new rotation concerning different methods

(Note: Box plot data are human current averaged over different day from 2022/3 to 2022/11, time period is from 5 am to 24 pm per 30 minutes, locations is about 30,000 nodes in the greater Tokyo area, sample size is the number of locations times time period, which is about 1,114,000)

Fig.12 shows the CDF of rotation calculated by different methods. It is meaningless to compare the rotation value calculated by different methods directly. Thus, we make the mean value of rotation calculated by all methods consistent and then compare. As a result, there is still not much difference between rotation calculated by different new methods with respect to distribution.


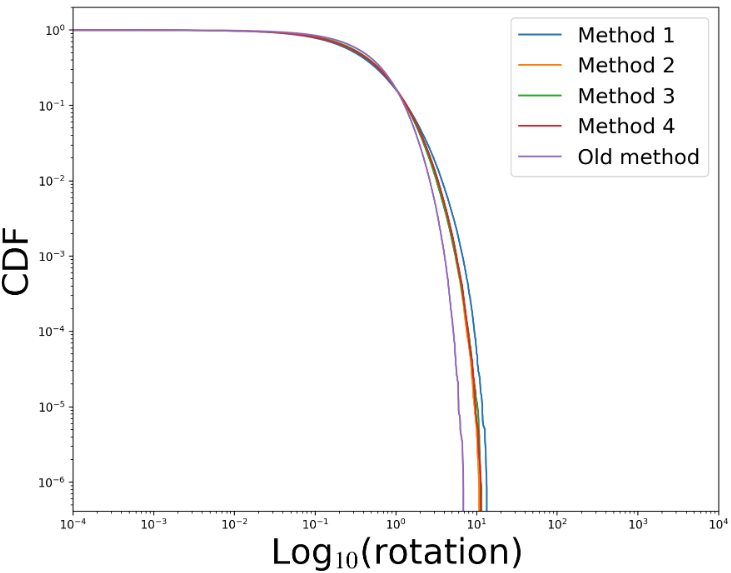


Figure 12. Comparison of CDF of rotation calculated in different methods

Moreover, we tried to plot the value of the loss function (Eq.(2)) of Adam concerning the different time periods in a day. As shown in Fig.13, the purple colour line (0 iterations) drops to the brown colour line after the 2000 iteration and drops to the pink one after the 10,000 iterations. The value of the loss function decrease, and the time series becomes steadier. We tried to compare the trend of the time series, and we found that the trend of our case (red colour line with rectangle) is close to the one after the 2000 iteration.


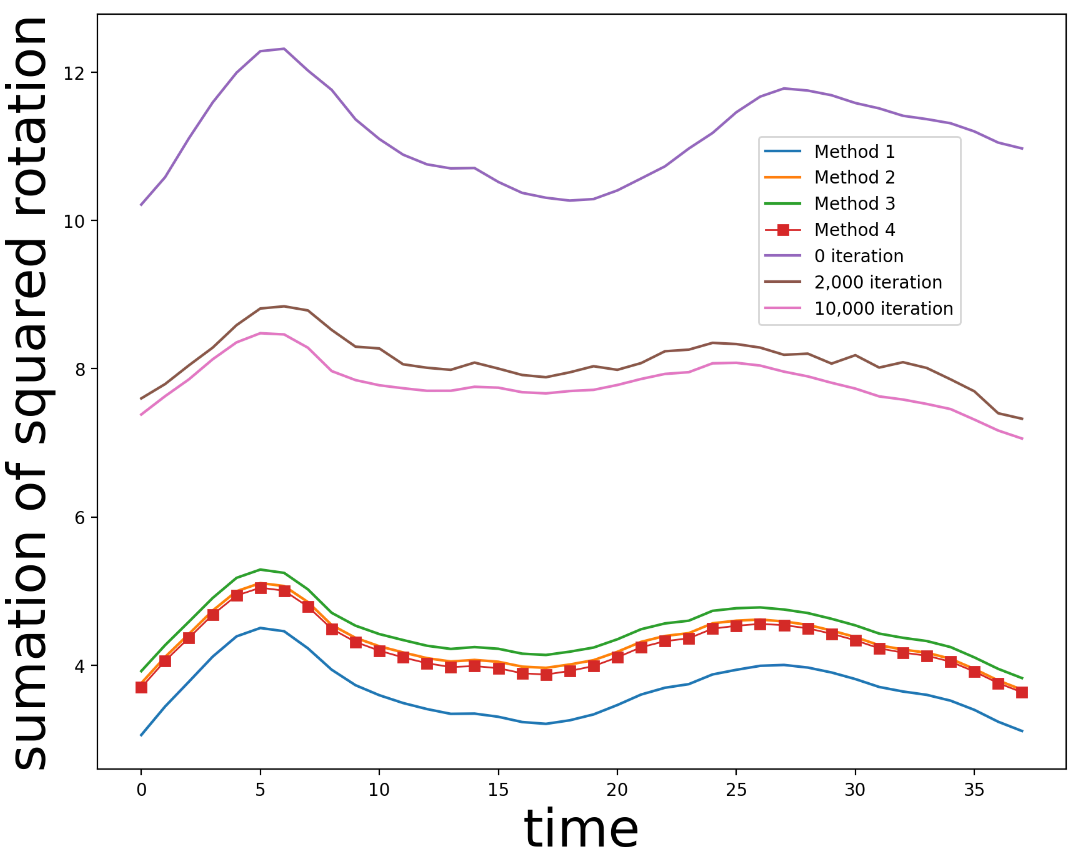


Figure 13. Comparison of loss function when calculating resistance concerning different methods (Note: line with rectangle is the method recommended in our study)

In Fig.14, rotation spatial distribution shows we also achieve removing rotation of electric field in electric circuit as much as possible in rush hour (left), and there is almost no rotation during other time period (right).


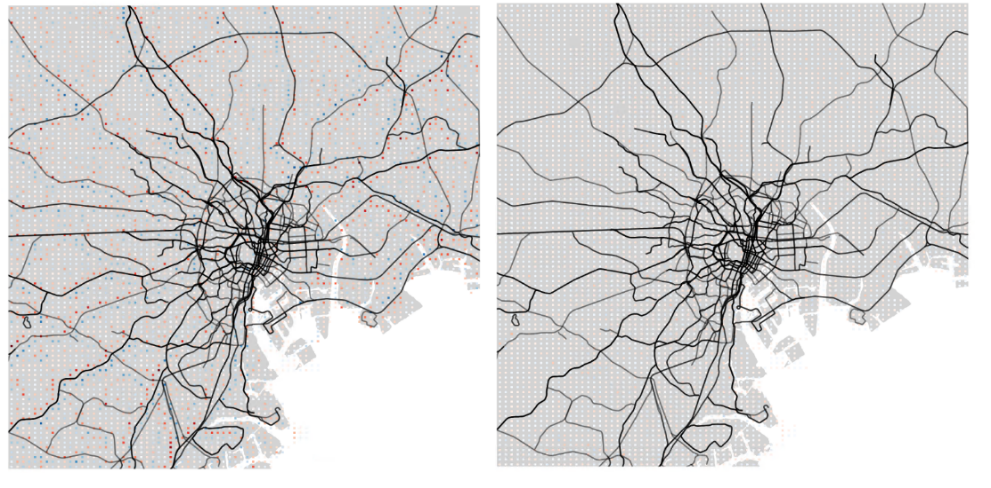


Figure 14. Rotation spatial distribution calculated by method recommended by us (Note: left is from 7:30 to 8:00 rush hour, right is from 13:30 to 14:00)

In Fig.15, when time period is fixed to 14:00 pm at which human current is minimum with a day, variant of current among different day keeps a linear relation with human conductivity, the inverse of human resistance, which is also consistant with the result claimed in the previous research, even though we changed the method to calculate resistance value.


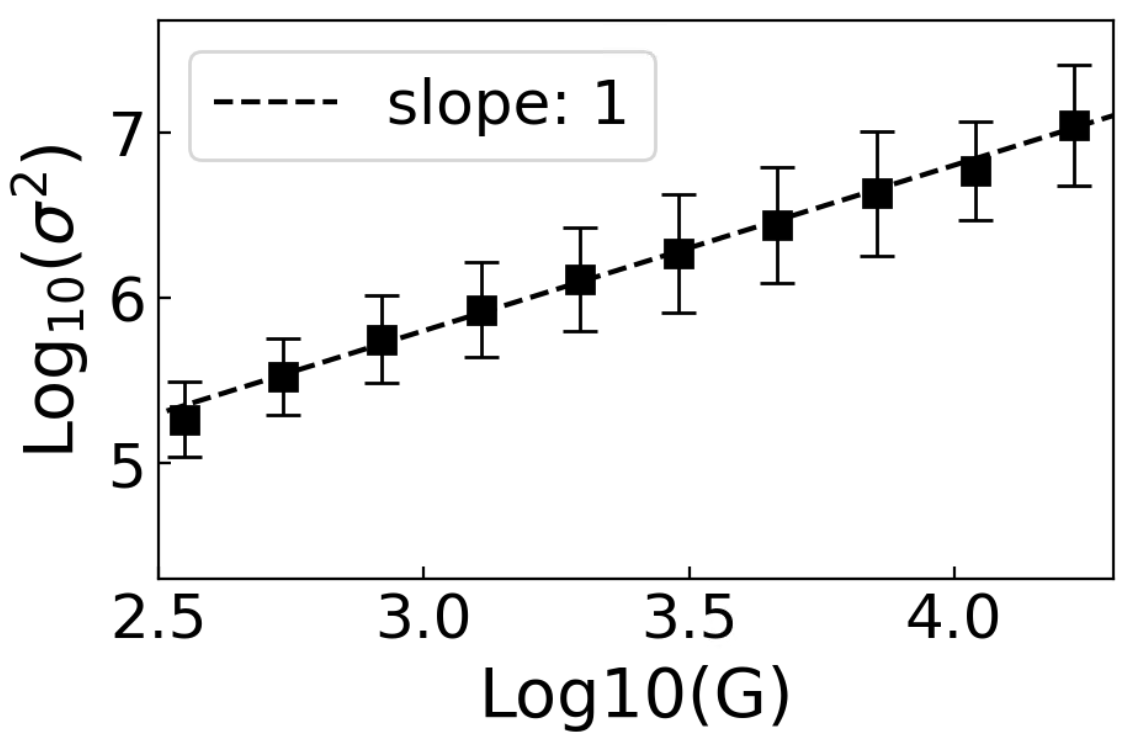


Figure 15. Relation between conductivity and variant of current

Overall, we consider that our new methods can replace the old method in the previous research to calculate human potential because it shows that new and old potential keep a well-linear relationship in Fig.4. And our new methods solved the limitation of the previous method, including high computational cost, results being not unique, hard to deploy and use due to fine-tuning of hyperparameters, and calculation error (outlier) in rural areas, as told in 2.2. Besides, there is not much difference between the property of new and old methods proposed new methods. Therefore, people can choose the suitable method to calculate resistance and human potential.

# **2.6 Conductivity spatial distribution on different cities in Japan**


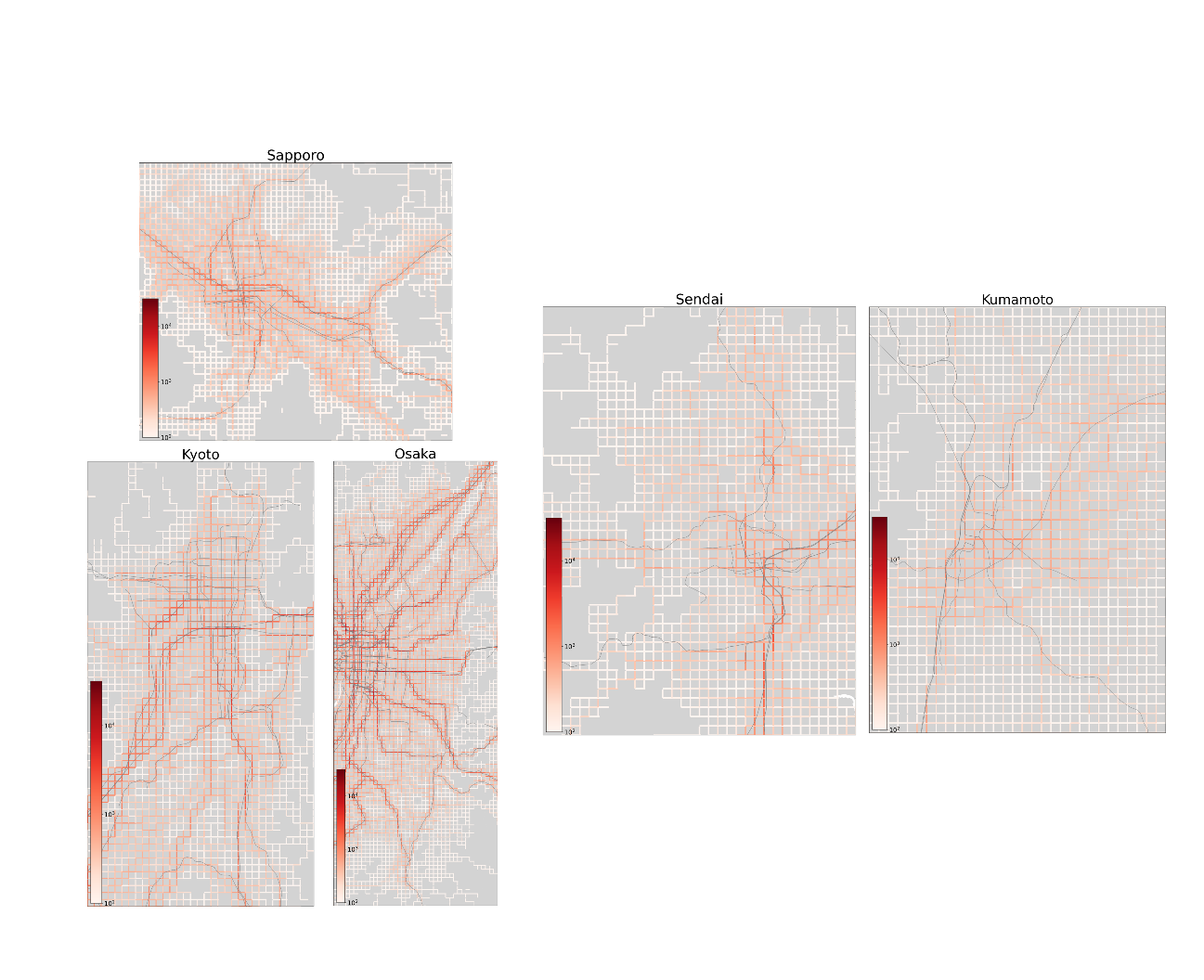
Figure 16. Conductivity spatial distribution under different cities

Fig.16 shows human conductivity spatial distribution on different cities in Japan, including Kyoto, Osaka, Sendai, Sapporo, and Kumamoto. Generally, strong conductivity is observed near the railway line (grey lines in figure) in metropolitan such as Osaka. Nevertheless, in cities with less railway, car will become the main transportation tool and conductivity will reflect the total trip demand.

# **3. Route Generation Model**

In this section, we will propose our recommended method (using current mean value) to determine resistance in 3.1 and show that there are three advantages to doing so. Then, we show that the result of RGM does not rely on the system's boundary in 3.2, so that people can select any observation area that is interested in generating the route. Finally, we will give more examples of the route generated by RGM in 3.3.

# **3.1 The method we recommend using to determine resistance**

Section 2 showed there are not much difference between different methods to calculate resistance, including different quantile currents and mean current values. In this section, we recommend that using the mean absolute current value is the best way to calculate resistance. There are three reasons for it.

The first reason is that according to LLN (Law of Large Numbers), the mean value will converge to the expectation of the exponential distribution and become steadier and more robust with the increase of data. The resistance is supposed to reflect the infrastructure level so the value should be determined as robust as possible from the data.

The second reason is that we can implement real-time online updates for resistance value. Given the old mean current value ${MI}_{old}$ and the old number of samples $N_{old}$, when data of human current of the next day $I_{new}$ comes, the new mean current value ${MI}_{new}$ of a place can be updated instantly as follows.

${MI}_{new} =\frac{{MI}_{old}*N_{old}+I_{new}}{N_{new}}$ (13)

where, $N_{new}=N_{old}+1$

This method's time and space complexity both is $O(1)$ because we only need to maintain and update two variables $MI$ and $N$. While updating per cent quantile current requires maintaining an array when new data comes, sorting the array and finding a specific per cent quantile current value. The time complexity of sort algorithms is $O(n^{2}$) or $O(n*log n$) depend on different array properties. If the user maintains a sorted array, it still spends $O(n$) to search a certain per cent quantile number or spends $O(log n$) to search it by using binary search for an ordered array. Moreover, the space complexity of the latter is also $O(n)$. Therefore, using mean value can make the time and space complexity of real-time online update $O(1)$, which is independent of the size of system $n$ and more suitable to be applied to big data.

The third reason is that mean current has better interpretability for human resistance. In the previous research, optimization algorithm was like a black box, and it is hard to therotically parse the meaning or property of calculated human resistance. However, in our study, in the main text of this paper, we have the following Eq. (14-16):

$I_{(d,t,L,\sigma)}=\frac{\left( v_{(d,t,L,\sigma)}*p_{(d,t,L)}+v_{(d,t,\sigma\left( L \right),\sigma)}*p_{(d,t,\sigma\left( L \right))} \right)}{2}$ (14)

$R_{(L,\sigma)}:=\frac{1}{\underset{d,t}{\mathrm{mean}} \left\{ |I_{(d,t,L,\sigma)}| \right\}}$ (15)

$\rho_{(L,\sigma)}=1/\min_{dir} \{R_{(L=des,\sigma)}\}$ (16)

Eq.(17) can be obtained by combining Eq.(14-16)

$\rho_{(L,\sigma)}=\frac{1}{R_{(L,\sigma)}}=\underset{d,t}{\mathrm{mean}} \left\{ |I_{(d,t,L,\sigma)}| \right\}=\underset{d,t}{\mathrm{mean}} \left\{ \frac{\left( v_{(d,t,L,\sigma)}*p_{(d,t,L)}+v_{(d,t,\sigma\left( L \right),\sigma)}*p_{(d,t,\sigma\left( L \right))} \right)}{2} \right\}$ (17)

Where $\sigma$∈{$\sigma_{+x},\sigma_{+y}, \sigma_{-x},\sigma_{-y}$}

We explain why conductivity reflects infrastructure level in the following text. When the population $p_{(d,t,L)}$ in node $L$ is fixed, the faster people move in an area, the larger $v_{(d,t,L)}$ (mean average velocity for every people in node $L$, date *d*, time period *t*), means this area has better transportation tools. For example, according to the moving speed, transportation can be roughly classified into four classes: walking (below 10 km/h), bus (10 km/h – 80 km/h), subway and train (80 km/h – 100 km/h), and high-speed train (200 km/h – 250 km/h). Conversely, if the average moving speed of every people in a node $v_{(d,t,L)}$ is fixed, higher $p_{(d,t,L)}$ means higher population density that the node can transport, which is also reflecting the infrastructure level. (We determined the resistance value using the mean current value for the three reasons above)

# **3.2 Boundary selection of RGM**

In the main text of our paper, we suggest that people can determine the number of nodes and the shape of the boundary in RGM arbitrarily, satisficing the following two conditions, 1) only one connected component in the electric circuit network, and 2) the network include the origin node and destination node. The first condition must be satisfied because a possible route must exist from the origin to the destination.


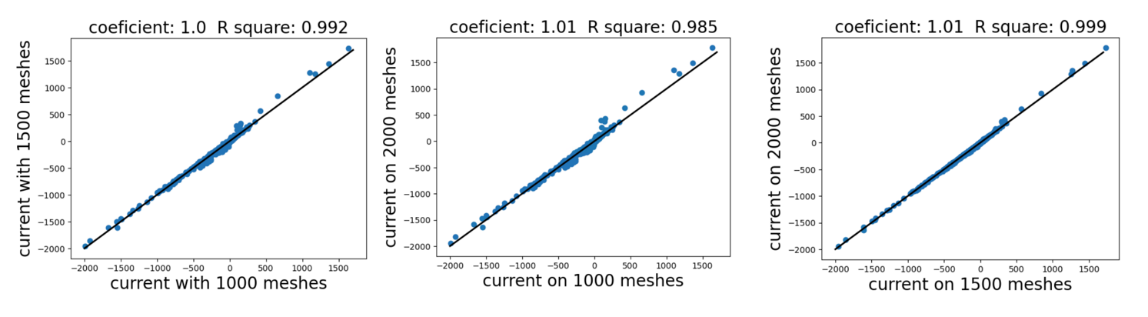


Figure 17. Relationship between different size of system. (Note: x-axis and y-axis are the comparisons between generated current value when network contains 1500, 2000, and 2500 nodes, respectively)

As shown in Fig.17, we make the observation area larger (change the network size) and run RGM (the origin and the destination are the same as the places mentioned in the body text). It is found that the solution of RGM in different network size keep a well-linear relationship (slope of linear regression is 1), which means the solution does not rely on the boundary that people set, and the boundary can be to any convenient shape for computating.

# **3.3 More examples of route generation**

We run our RGM and found our model works well on other places on the map, as shown in Fig. (18-19). Our model generates recommended routes provided by Google Maps. In rural areas, generated roads will be relatively simple because people can only go the highway or the railway, which is close to 1-dimension. In urban areas, generated roads will be relatively complicated because there are too many available routes to the destination, which is close to 2-dimension.


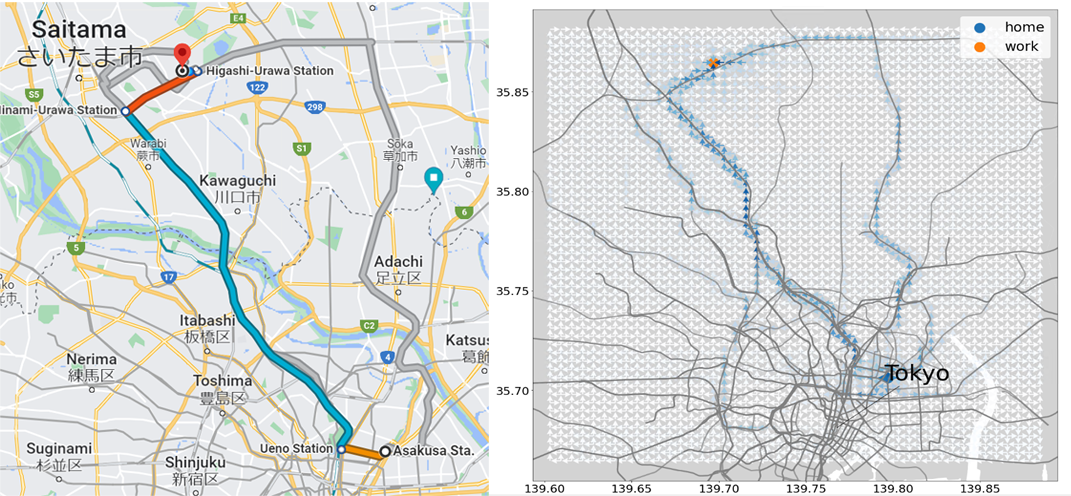


Figure 18. RGM example 1 (origin: Asakusa station, destination: Higashi-Urawa station, left picture cited from Google Map^2^)


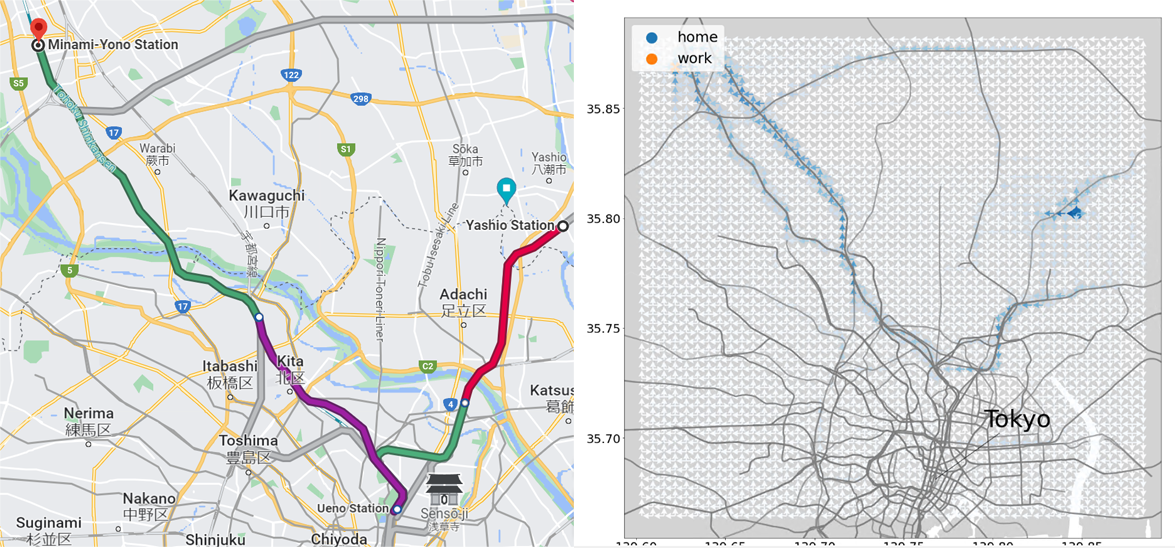


Figure 19. RGM example 2 (origin: Yashio station, destination: Minami-Yono station, left picture cited from Google Map^3^)

As shown in Figure 20, when Tama monorail was blocked in the left of Tokyo, people will bypass that area to go to the destination.


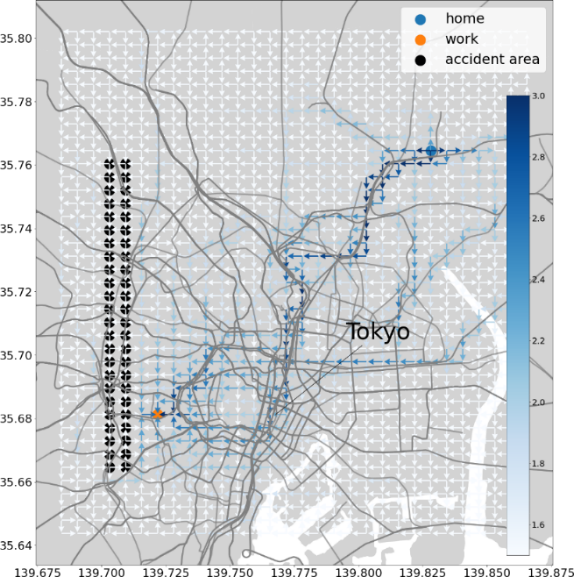


Figure 20. Route generation by RGM (grey line: railway line, black dots: accident area near Tama monorail, example 2, home: where people live, work: where people go to work)

# **4. Review of the method of calculating human potential**

In the previous research^1^, a detailed way to calculate electric potential on a map is not shown clearly. Therefore, as a supplement, we want to propose our procedure to calculate the value of potential in detail.

Firstly, we need to calculate the charge $Q_{(d,t,L)}$, the divergence of voltage, at date *d*, time period *t* and location $L$.

$Q_{(d,t,L)}=div\left( I_{d,t,L}*R_{L} \right)=\sum_{i=1}^{4} I_{(d,t,L,\sigma_{i})}*R_{(L,\sigma)}$ (18)

The map's mesh number is fixed when the observed data are given. In this case, we can view each mesh as a node in the 2-dimensional square lattice and define the adjacent matrix for each node. First, find the network's largest connected component by Breadth-First Search algorithm. Then, we define the adjacent matrix:

$A_{i,j}=\left\{ \begin{aligned} 1, &node i is adjacent to j \\ 0, &else \end{aligned} \right.$ (19)

Moreover, we define the diagonal matrix, reflecting how many nodes are adjacent nearby, for the node on the sea boundary (sea boundary is defined by using land use data that the sea ratio of a node above 95% will be viewed as a sea area. The data was statistics by the Japan government):

$D_{i,j}=\left\{ \begin{aligned} \sum_{j} A_{i,j} , case 1 \\ 4, case 2 \\ 0, case 3 \end{aligned} \right.$ (20)

where, $case 1:i=j and node i is adjacent to sea area$

$case 2: i=j and node i is on the other area$

$case 3: i\neq j$

Theoretically, a boundary condition that $\emptyset=0$ near the land boundary needs to be set, but practically, we only need to solve human potential $\emptyset_{d,t,l}$ at day *d*, time period *t*, location $L$ by solving the following formula. The number of variables and linear equations is the number of nodes (locations).

$\left( D_{i,j}-A_{i,j} \right)*\left( \begin{matrix} \emptyset_{d,t,1} \\ \ldots\\ \emptyset_{d,t,L} \end{matrix} \right)=\left( \begin{matrix} div\left( I_{d,t,1}*R_{1} \right) \\ \ldots\\ div\left( I_{d,t,L}*R_{L} \right) \end{matrix} \right)$ (21)

Matrix $\left( D_{i,j}-A_{i,j} \right)$ also called Laplacian matrix $\Delta_{i,j}$ in a network. The time complexity of BFS is $O(V+E$), where $V$ is the number of vertices and $E$ is the number of edges. In our case, $V$ equals $L,$ and *E* approximately equals$4*L$ because it is a lattice network. Therefore time complexity of BFS is $O(4*L+ L$)→$O(L$).

The time complexity of calculating charge and solving linear equations is $O(L*d*t$) and $O(L^{3}$) respectively, therefore the total time complexity of calculating potential is $O(L)+O(L*d*t)+O(L^{3})\to O(L^{3})$. Similar to the discussion part in main text, we can reduce the computational cost to $O(L^{2.33})$ practically, when the coefficient matrix is very sparse^5^.

# **5. Brief introduction of our open-source code**

Users can download open source computer programming language, called Python 3, to use our code to conduct research about human flow pattern. The code related to RECM will be provided as a Python script file in the following URL: <https://github.com/Zhihua-Zhong/Revised-Electric-Circuit-Model>. The method to calculate human current, human resistance and other variables has been encapsulated into Python functions that can be called and customized by other programmers and users. People with input data that fulfils the standard input data format shown in Fig. 1 can use our codes to implement RECM directly. If not, a simple format transformation is required beforehand. Regarding the development environment, python 3.8.5 with third-party dependencies, including pandas, geopandas, numpy, matplotlib, and scipy are required. More details and hand-to-hand tutorials can be found on our GitHub through the above URL.

In order to increase computing efficiency, multi-process calculation functions were also developed to enable users with powerful computers to use multi CPUs to compute the result simultaneously. Specifically speaking, because the calculation of human current and resistance is mutually independent at different nodes and independent on different days, the calculation on different spaces and times can be separated and paralleled to the different CPUs.

Our open-source code will be updated over time and new function will be gradually online to respond to other researchers' new demands. If there is any query, please do not hesitate to contact us through the contact information revealed in the above website.

# **Reference:**

[1]. Shida, Y., Ozaki, J., Takayasu, H. et al. Potential fields and fluctuation-dissipation relations derived from human flow in urban areas modeled by a network of electric circuits. Sci Rep 12, 9918 (2022).

[2]. Google Map. Recommendation route from Asakusa station to Higashi-Urawa station. 2023. https://www.google.com/maps/dir/Asakusa+Sta.,+1+Chome-1-3+Asakusa,+Taito+City,+Tokyo+111-0032/Higashi-Urawa+Station,+1+Chome+Higashiurawa,+Midori+Ward,+Saitama/@35.7924717,139.6410068,11z/data=!4m15!4m14!1m5!1m1!1s0x60188ec690127e2f:0xf41f021a3a02cff0!2m2!1d139.7977621!2d35.7107924!1m5!1m1!1s0x601895476d3d457f:0x7efa978a2dc696f8!2m2!1d139.7046531!2d35.8641332!3e3!5i1?entry=ttu

[3]. Google Map. Recommendation route from Yashio station to Minami-Yono station. 2023. https://www.google.com/maps/dir/Yashio+Station,+6+Chome+Oze,+Yashio,+Saitama/Minami-Yono+Station,+2+Chome+Suzuya,+Chuo+Ward,+Saitama/@35.7940843,139.7671473,11z/data=!3m1!4b1!4m14!4m13!1m5!1m1!1s0x6018900d65a25555:0x46f85833948553da!2m2!1d139.8448262!2d35.8078355!1m5!1m1!1s0x6018c1c1b61dc479:0x84798c2ff5215cdb!2m2!1d139.6311953!2d35.8674942!3e3?entry=ttu

[4]. Kingma, Diederik P., and Jimmy Ba. "Adam: A method for stochastic optimization." arXiv preprint arXiv:1412.6980 (2014).

[5]. Peng, Richard, and Santosh Vempala. "Solving sparse linear systems faster than matrix multiplication." In Proceedings of the 2021 ACM-SIAM symposium on discrete algorithms (SODA), pp. 504-521. Society for Industrial and Applied Mathematics, 2021.
